# Supplementary material for: A history of olive and grape cultivation in Southwest Asia using charcoal and seed remains
Source: PLoS One. 2024 Jun 20;19(6):e0303578. doi: 10.1371/journal.pone.0303578 (PMC11189204; doi:10.1371/journal.pone.0303578)
Supplement: S1 Table — Percentages of olive/grape seeds among the crops and ubiquities among the samples, as well as charcoal percentages and total number of charcoals are given for each site. (DOCX) [file pone.0303578.s001.docx]

| **site name** | **Latitutde** | **Longitude** | **Region** | **Start occupation BC** | **End occupation BC** | **Average occupation BC** | **Period BC** | **Elevation (m)** | **modern rainfall. hindcasting (mm)** | **reconstructed rainfall. hindcasting (mm)** | ***MINIMUM* reconstrructed rainfall. hindcasting (mm)** | ***MAXIMUM* reconstrructed rainfall. hindcasting (mm)** | ***Vitis* seed % of crops** | ***Vitis* seed ubiquity %** | ***Olea* stones % of crops** | ***Olea* stone ubiquity %** | ***Olea* charcoal %** | ***Vitis* charcoal %** | **Number/ml/gr of charcoal fragments** | **Reference seeds** | **Reference charcoal** |
| --- | --- | --- | --- | --- | --- | --- | --- | --- | --- | --- | --- | --- | --- | --- | --- | --- | --- | --- | --- | --- | --- |
| Tell Sabi Abyad | 36.504 | 39.090 | Mesopotamia | 6400 | 5800 | 6100 | 6500-3600 | 330 | 365 | 396 | 293 | 474 | 0.000 | 0.000 | 0.000 | 0.000 |  |  |  | (1) |  |
| Nahal Zippori 3 / Tell Mitzpe Zevulun North | 32.746 | 35.279 | Southern Levant | 6400 | 5800 | 6100 | 6500-3600 | 260 | 666 | 724 | 535 | 865 | 0.000 | 0.000 | 0.000 |  | 0.00 | 0.00 | 297 | (2) | (2) |
| Shaʿar Hagolan | 32.686 | 35.603 | Southern Levant | 6400 | 5800 | 6100 | 6500-3600 | -192 | 511 | 555 | 411 | 664 | 0.000 | 0.000 | 0.280 | 3.000 |  |  |  | (3) |  |
| Jericho | 31.850 | 35.436 | Southern Levant | 6000 | 5600 | 5800 | 6500-3600 | -209 | 447 | 485 | 359 | 579 | 0.000 | 0.000 | 0.000 | 0.000 |  |  |  | (4) |  |
| Nahal Zehora II | 32.550 | 35.118 | Southern Levant | 6400 | 5200 | 5800 | 6500-3600 | 241 | 676 | 735 | 544 | 878 | 0.000 | 0.000 | 0.028 |  | 5.00 | 0.00 | 63 | (5) | (6) |
| Fistikli Höyük | 37.058 | 37.885 | Mesopotamia | 5850 | 5700 | 5775 | 6500-3600 | 346 | 510 | 554 | 409 | 661 | yes |  | yes |  |  |  |  | (7) |  |
| Umm Qseir | 36.406 | 40.849 | Mesopotamia | 6000 | 5300 | 5650 | 6500-3600 | 287 | 339 | 368 | 272 | 439 | 0.000 | 0.000 | 0.000 | 0.000 |  |  |  | (8) |  |
| Tell el'Oueili | 31.220 | 45.870 | Mesopotamia | 6500 | 4500 | 5500 | 6500-3600 | 8 | 108 | 117 | 87 | 140 | 0.000 | 0.000 | 0.000 | 0.000 |  |  |  | (9) |  |
| Ras Shamra | 35.602 | 35.785 | Northern Levant | 5750 | 5250 | 5500 | 6500-3600 | 26 | 772 | 839 | 620 | 1002 | 0.030 | 0.480 | 0.200 | 40.300 |  |  |  | (10) |  |
| Tell Aqab | 37.058 | 40.896 | Mesopotamia | 5500 | 5300 | 5400 | 6500-3600 | 448 | 432 | 470 | 347 | 561 | 0.000 | 0.000 | 0.000 | 0.000 |  |  |  | (8) |  |
| Tepe Sialk | 33.969 | 51.405 | Iran | 6400 | 4200 | 5300 | 6500-3600 | 956 | 158 | 172 | 127 | 205 | 0.000 | 0.000 | 0.000 | 0.000 | 0.00 | 0.00 | 400 | (11) | (12) |
| Choga Mami | 33.730 | 45.520 | Mesopotamia | 6500 | 4100 | 5300 | 6500-3600 | 26 | 231 | 252 | 186 | 300 | 0.000 | 0.000 | 0.000 | 0.000 |  |  |  | (13) |  |
| Tell Kurdu | 36.330 | 36.445 | Northern Levant | 6000 | 4500 | 5250 | 6500-3600 | 89 | 547 | 594 | 440 | 710 | 0.000 | 0.000 | 0.000 | 0.000 |  |  |  | (14, 15) |  |
| Kosak Shamali | 36.558 | 38.280 | Mesopotamia | 5300 | 5000 | 5150 | 6500-3600 | 325 | 403 | 438 | 324 | 524 | 0.000 | 0.000 | 0.000 | 0.000 | 0.00 | 0.00 | 357 | (16) | (17) |
| Jaffarabad | 32.180 | 48.450 | Iran | 6000 | 4000 | 5000 | 6500-3600 | 86 | 296 | 322 | 238 | 384 | 0.000 | 0.000 | 0.000 | 0.000 |  |  |  | (18) |  |
| Tawila | 36.540 | 39.494 | Mesopotamia | 5850 | 4000 | 4925 | 6500-3600 | 368 | 366 | 398 | 294 | 475 | 0.000 | 0.000 | 0.000 | 0.000 | 0.00 | 0.00 | 1995 | (19) | (19) |
| Ras Shamra | 35.602 | 35.785 | Northern Levant | 5250 | 4300 | 4775 | 6500-3600 | 26 | 772 | 839 | 620 | 1002 | 0.020 | 18.200 | 0.020 | 45.400 |  |  |  | (10) |  |
| Tepe Hasanlu region | 37.000 | 45.470 | Iran | 6400 | 3100 | 4750 | 6500-3600 | 1305 | 393 | 427 | 316 | 510 | yes |  | 0.000 | 0.000 |  |  |  | (20) |  |
| Tel Tsaf | 32.170 | 35.430 | Southern Levant | 5000 | 4500 | 4750 | 6500-3600 | -259 | 558 | 606 | 448 | 724 | 0.000 | 0.000 | yes |  | 8.10 | 0.00 | 643 | (21) | (22, 23) |
| Tell esh-Shuna | 32.611 | 35.612 | Southern Levant | 5100 | 4750 | 4925 | 6500-3600 | -208 | 499 | 542 | 401 | 648 | 0.000 | 0.000 | 0.797 | 9.000 | 99.00 |  |  | (24) | (25) |
| Surezha | 39.956 | 39.844 | Mesopotamia | 5200 | 3800 | 4500 | 6500-3600 | 1957 | 533 | 579 | 428 | 692 | 0.000 | 0.000 | 0.000 | 0.000 | 0.00 | 0.00 | 105 | (26) | (26) |
| Bendebal | 32.330 | 48.270 | Iran | 4700 | 4200 | 4450 | 6500-3600 | 92 | 309 | 336 | 248 | 401 | 0.000 | 0.000 | 0.000 | 0.000 |  |  |  | (18) |  |
| Tepe Farukhabad | 32.587 | 47.224 | Iran | 5100 | 3800 | 4450 | 6500-3600 | 140 | 260 | 282 | 209 | 337 | 0.000 | 0.000 | 0.000 | 0.000 |  |  |  | (27) |  |
| Pella | 32.450 | 35.613 | Southern Levant | 5100 | 3700 | 4400 | 6500-3600 | -37 | 482 | 524 | 388 | 626 | 0.000 | 0.000 | 1.866 | 100.000 |  |  |  | (28) | (29) |
| Abu Hamid | 32.320 | 35.550 | Southern Levant | 4800 | 3800 | 4300 | 6500-3600 | -211 | 480 | 521 | 385 | 622 | 0.000 | 0.000 | yes | 0.000 | 67.50 | 0.00 | n.a. | (30) | (30) |
| Tilbeshar | 36.873 | 37.559 | Mesopotamia | 5400 | 3100 | 4250 | 6500-3600 | 604 | 535 | 581 | 429 | 694 | 1.709 |  | 0.000 | 0.000 |  |  |  | (31) |  |
| Tell Ilbol | 36.595 | 37.189 | Northern Levant | 5300 | 3100 | 4200 | 6500-3600 | 471 | 472 | 513 | 379 | 612 | 0.000 | 0.000 | 0.000 | 0.000 |  |  |  | (32) |  |
| Nahal Qanah Cave | 32.150 | 35.100 | Southern Levant | 4700 | 3700 | 4200 | 6500-3600 | 394 | 643 | 699 | 517 | 834 | 0.000 | 0.000 | yes |  | yes |  |  | (33) | (22) |
| Shoham (North) | 31.997 | 34.952 | Southern Levant | 4700 | 3700 | 4200 | 6500-3600 | 96 | 583 | 634 | 469 | 757 | 0.000 | 0.000 | yes | 100.000 | 78.00 | 0.00 | 73 | (34) | (22) |
| Tuleilat Ghassul | 31.861 | 35.641 | Southern Levant | 4550 | 3850 | 4200 | 6500-3600 | -290 | 366 | 398 | 294 | 475 | 0.000 | 0.000 | 1.851 | 75.000 |  |  |  | (28) |  |
| Sataf | 31.769 | 35.125 | Southern Levant | 4700 | 3700 | 4200 | 6500-3600 | 597 | 614 | 667 | 493 | 796 | 0.000 | 0.000 | yes | 17.000 |  |  |  | (35) |  |
| Tell Abu Matar/Tel Beersheba | 31.230 | 34.780 | Southern Levant | 4700 | 3700 | 4200 | 6500-3600 | 256 | 297 | 323 | 239 | 386 | 0.000 | 0.000 | 0.000 | 0.000 |  |  |  | (36) |  |
| Tepe Yahya | 28.331 | 56.867 | Iran | 4500 | 3800 | 4150 | 6500-3600 | 1513 | 234 | 255 | 188 | 304 | yes | 50.000 | 0.000 | 0.000 |  |  |  | (37) |  |
| Hirbet el-Msas (Tel Masos) | 31.213 | 34.967 | Southern Levant | 4500 | 3700 | 4100 | 6500-3600 | 357 | 318 | 346 | 256 | 413 |  |  | yes |  | yes |  |  | (38) | (38) |
| Shiqmim | 31.130 | 34.700 | Southern Levant | 4500 | 3700 | 4100 | 6500-3600 | 258 | 231 | 251 | 186 | 300 | 0.000 | 0.000 | 0.000 | 0.000 |  |  |  | (39) |  |
| Tell Halif | 31.383 | 34.867 | Southern Levant | 4500 | 3700 | 4100 | 6500-3600 | 483 | 391 | 425 | 314 | 507 | 0.000 | 0.000 | 0.000 | 0.000 |  |  |  | (40) |  |
| Tell Chragh | 35.208 | 45.806 | Mesopotamia | 4500 | 3600 | 4050 | 6500-3600 | 545 | 425 | 462 | 342 | 552 | 0.000 | 0.000 | 0.000 | 0.000 |  |  |  | (41) |  |
| Tell Afis | 35.905 | 36.799 | Northern Levant | 4200 | 3800 | 4000 | 6500-3600 | 360 | 356 | 387 | 286 | 463 | 0.102 | 4.000 | 7.637 | 32.000 |  |  |  | (42) |  |
| Grai Resh | 36.318 | 41.919 | Mesopotamia | 4300 | 3600 | 3950 | 6500-3600 | 26 | 400 | 435 | 322 | 519 | 0.100 |  | 0.000 | 0.000 |  |  |  | (43) |  |
| Tappeh Gijlar | 37.930 | 45.300 | Iran | 4500 | 3100 | 3800 | 6500-3600 | 1267 | 345 | 374 | 277 | 447 | 0.000 | 0.000 | 0.000 | 0.000 |  |  |  | (44) |  |
| Korucutepe | 38.467 | 39.550 | Mesopotamia | 4500 | 3100 | 3800 | 6500-3600 | 820 | 543 | 590 | 436 | 704 | 0.000 | 0.000 | 0.000 | 0.000 | 0.00 | 0.00 | 33 | (45) | (45) |
| Tell Brak | 36.667 | 41.058 | Mesopotamia | 4000 | 3600 | 3800 | 6500-3600 | 360 | 376 | 409 | 302 | 488 | yes. no %; 0 |  | 0.000 | 0.000 | 0.00 | 0.20 | 1163 | (46, 47)* | (48) |
| Tell Hammam et-Turkman | 36.483 | 39.057 | Mesopotamia | 4500 | 3100 | 3800 | 6500-3600 | 339 | 365 | 397 | 293 | 474 | 0.000 | 0.000 | 0.000 | 0.000 |  |  |  | (49) |  |
| Oylum Höyük | 36.699 | 37.179 | Northern Levant | 4500 | 3100 | 3800 | 6500-3600 | 607 | 504 | 548 | 405 | 654 | 0.000 | 0.000 | 0.000 | 0.000 |  |  |  | (50) |  |
| Cayboyu (Asvan) | 38.917 | 38.950 | Mesopotamia | 4500 | 3100 | 3800 | 3600-3000 | 820 | 448 | 403 | 224 | 498 | 0.000 | 0.000 | 0.000 | 0.000 | 0.00 | 0.00 | n.a. | (51) | (52) |
| Tell Mozan | 37.057 | 40.997 | Mesopotamia | 3900 | 3600 | 3750 | 6500-3600 | 471 | 434 | 471 | 349 | 563 | 0.000 | 0.000 | 0.000 | 0.000 |  |  |  | (53) |  |
| Hamoukar | 36.812 | 41.956 | Mesopotamia | 4400 | 3000 | 3700 | 6500-3600 | 387 | 433 | 471 | 348 | 562 |  |  |  |  | 0.00 | 0.00 | 592 |  | (54) |
| Yarim Höyük | 37.025 | 37.970 | Mesopotamia | 3900 | 3100 | 3500 | 3600-3000 | 342 | 506 | 454 | 253 | 562 | 0.000 | 0.000 | 0.000 | 0.000 |  |  |  | (55) |  |
| Sharafabad | 32.050 | 48.780 | Iran | 3600 | 3300 | 3450 | 3600-3000 | 94 | 285 | 256 | 143 | 317 | 0.000 | 0.000 | 0.000 | 0.000 |  |  |  | (56) |  |
| Tell Brak | 36.667 | 41.058 | Mesopotamia | 3600 | 3300 | 3450 | 3600-3000 | 360 | 376 | 338 | 188 | 418 | yes; 0 |  | 0.000 | 0.000 | 0.00 | 0.00 | 812 | (46, 57) | (48) |
| Ashkelon | 31.664 | 34.547 | Southern Levant | 3600 | 3300 | 3450 | 3600-3000 | 18 | 428 | 385 | 214 | 476 |  |  |  |  | 85.10 | 0.00 | 174 |  | (58) |
| Afula | 32.611 | 35.290 | Southern Levant | 3600 | 3300 | 3450 | 3600-3000 | 65 | 613 | 550 | 306 | 680 | 0.000 | 0.000 | 0.000 | 0.000 |  |  |  | (59) |  |
| Sataf | 31.769 | 35.125 | Southern Levant | 3600 | 3300 | 3450 | 3600-3000 | 597 | 614 | 551 | 307 | 682 | 0.000 | 0.000 | yes |  |  |  |  | (35) |  |
| Wadi Fidan 4_E | 30.660 | 35.394 | Southern Levant | 3600 | 3300 | 3450 | 3600-3000 | 46 | 5 | 5 | 3 | 6 | 6.261 | 90.000 | 0.000 | 0.000 |  |  |  | (60) |  |
| Tell Jerablus Tahtani | 36.790 | 38.021 | Mesopotamia | 3600 | 3100 | 3350 | 3600-3000 | 337 | 458 | 412 | 229 | 509 | 0.000 | 0.000 | 0.000 | 0.000 | 0.00 | 0.30 | 305 | (61, 62) | (63) |
| Hacinebi Tepe | 34.556 | 36.519 | Northern Levant | 3600 | 3100 | 3350 | 3600-3000 | 519 | 512 | 460 | 256 | 568 | 0.000 | 0.000 | 0.000 | 0.000 |  |  |  | (64, 65) |  |
| Jawa | 32.335 | 37.003 | Southern Levant | 3600 | 3100 | 3350 | 3600-3000 | 1040 | 161 | 144 | 80 | 178 | yes |  | 0.000 | 0.000 | 0.00 | 0.00 |  | (66) | (67) |
| Jericho | 31.850 | 35.436 | Southern Levant | 3600 | 3100 | 3350 | 3600-3000 | -209 | 447 | 401 | 223 | 496 | 0.000 | 0.000 | 0.000 | 0.000 | 0.00 | 0.00 | 58 | (4) | (68) |
| City of David_Jerusalem | 31.772 | 35.235 | Southern Levant | 3600 | 3100 | 3350 | 3600-3000 | 703 | 620 | 557 | 310 | 689 |  |  | yes |  |  |  |  | (69) |  |
| Tell esh-Shuna | 32.611 | 35.612 | Southern Levant | 3600 | 3100 | 3350 | 3600-3000 | -208 | 499 | 448 | 250 | 555 | 15.376 | 46.000 | 7.295 | 37.000 |  |  |  | (24) |  |
| Tell Fadous-Kfarabida | 34.226 | 35.660 | Northern Levant | 3600 | 3000 | 3300 | 3600-3000 | 21 | 873 | 785 | 437 | 970 | 18.182 | 33.000 | 54.545 | 67.000 | 42.10 | 0.00 | 166 | (70-72) | (54, 70, 71) |
| Megiddo | 32.585 | 35.185 | Southern Levant | 3600 | 3000 | 3300 | 3600-3000 | 162 | 644 | 579 | 322 | 716 | 0.000 | 0.000 | 32.787 | 33.000 | 48.90 | 0.00 | 92 | (73) | (74) |
| En Besor | 31.320 | 34.470 | Southern Levant | 3600 | 2900 | 3250 | 3600-3000 | 86 | 292 | 262 | 146 | 324 | yes |  | 0.000 | 0.000 | 0.00 | 0.00 | 41 | (75) | (76) |
| Tel Dalit_Khirbet Ras ed-Dhaliye | 31.771 | 34.969 | Southern Levant | 3600 | 2900 | 3250 | 3600-3000 | 348 | 557 | 500 | 278 | 618 | 0.000 | 0.000 | 97.619 |  |  |  |  | (77) | (77) |
| Tell el-Handaquq North | 32.302 | 35.597 | Southern Levant | 3600 | 2900 | 3250 | 3600-3000 | -158 | 468 | 421 | 234 | 520 | 0.000 | 0.000 | 0.000 | 0.000 |  |  |  | (78) |  |
| Tell Gezer | 31.860 | 34.920 | Southern Levant | 3600 | 2900 | 3250 | 3600-3000 | 26 | 550 | 494 | 275 | 611 | yes |  | yes |  |  |  |  | (75) |  |
| Horum Hüyük | 37.100 | 37.867 | Mesopotamia | 3300 | 3100 | 3200 | 3600-3000 | 359 | 522 | 469 | 261 | 580 |  |  |  |  | 0.00 | 0.02 | 5935 |  | (17) |
| Hassek Höyük | 37.750 | 38.917 | Mesopotamia | 3300 | 3100 | 3200 | 3600-3000 | 530 | 514 | 462 | 257 | 571 | 0.000 | 0.000 | yes | 5.000 |  |  |  | (79) |  |
| Ras an-Numayra | 31.131 | 35.525 | Southern Levant | 3300 | 3100 | 3200 | 3600-3000 | -349 | 123 | 111 | 62 | 137 | 19.597 |  | 0.092 |  |  |  |  | (80) |  |
| Tell Abu al-Kharaz | 32.399 | 35.595 | Southern Levant | 3300 | 3100 | 3200 | 3600-3000 | -133 | 479 | 430 | 240 | 532 | 1.252 | 50.000 | 0.063 | 13.000 |  |  |  | (81) |  |
| Tell el Ifshar | 32.372 | 34.908 | Southern Levant | 3300 | 3100 | 3200 | 3600-3000 | 20 | 670 | 602 | 335 | 744 | 0.000 | 0.000 | 0.000 | 0.000 |  |  |  | (82) |  |
| Tepe Hissar | 36.150 | 54.070 | Iran | 3400 | 2900 | 3150 | 3600-3000 | 1621 | 205 | 185 | 102 | 223 | 0.000 | 0.000 | 0.000 | 0.000 |  |  |  | (83) |  |
| Arad | 31.281 | 35.125 | Southern Levant | 3300 | 2900 | 3100 | 3600-3000 | 569 | 355 | 319 | 177 | 394 | 2.299 | 4.000 | 35.057 | 100.000 | 50.50 | 2.10 | 97 | (84) | (22, 85) |
| Tel Beth Yerah_Khirbet Kerak | 32.715 | 35.572 | Southern Levant | 3300 | 2900 | 3100 | 3600-3000 | -199 | 534 | 480 | 267 | 593 | 11.100 |  | 22.200 |  | yes |  |  | (86) | (86) |
| Bab'edh Dhra | 31.253 | 35.533 | Southern Levant | 3300 | 2900 | 3100 | 3600-3000 | -247 | 194 | 174 | 97 | 215 | 8.320 | 57.000 | 0.616 | 14.000 |  |  |  | (87) |  |
| Pella | 32.450 | 35.613 | Southern Levant | 3300 | 2900 | 3100 | 3600-3000 | -37 | 482 | 433 | 241 | 536 | 0.495 | 100.000 | 0.825 | 67.000 |  |  |  | (88) |  |
| Beth Shean | 32.504 | 35.503 | Southern Levant | 3200 | 2950 | 3075 | 3600-3000 | -120 | 508 | 457 | 254 | 565 | 0.192 | 55.000 | 0.057 | 36.000 | 33.30 | 0.00 | 27 | (89) | (90) |
| Tell Karrana | 36.717 | 42.917 | Mesopotamia | 3300 | 2800 | 3050 | 3600-3000 | 325 | 419 | 377 | 210 | 466 | 0.000 | 0.000 | 0.000 | 0.000 |  |  |  | (91) |  |
| Tell Halif | 31.383 | 34.867 | Southern Levant | 3600 | 2500 | 3050 | 3600-3000 | 483 | 391 | 351 | 195 | 434 | 0.000 | 0.000 | 0.000 | 0.000 |  |  |  | (40) |  |
| Malyan | 30.050 | 52.420 | Iran | 3400 | 2600 | 3000 | 3600-3000 | 1618 | 254 | 228 | 127 | 282 | 0.000 | 0.000 | 0.000 | 0.000 | 0.00 | 0.00 | 1620 | (92) | (92) |
| Tel Yarmouth/Tel Yarmut | 31.708 | 34.975 | Southern Levant | 3100 | 2900 | 3000 | 3600-3000 | 399 | 546 | 490 | 273 | 606 | 20.476 | 43.000 | 26.667 | 50.000 |  |  |  | (93) |  |
| Tell el-Fukhar | 32.589 | 35.953 | Southern Levant | 3100 | 2900 | 3000 | 3600-3000 | 434 | 392 | 352 | 196 | 435 | 0.000 | 0.000 | 0.000 | 0.000 |  |  |  | (94) |  |
| Tell Shiukh Fawqani | 36.767 | 38.050 | Mesopotamia | 3100 | 2900 | 3000 | 3000-2500 | 345 | 447 | 431 | 338 | 500 | 0.000 | 0.000 | 0.000 | 0.000 |  |  |  | (95) |  |
| Tell Abu al-Kharaz | 32.399 | 35.595 | Southern Levant | 3050 | 2900 | 2975 | 3000-2500 | -133 | 479 | 462 | 363 | 536 | 0.150 | 33.000 | 0.006 | 33.000 |  |  |  | (81) |  |
| Taskun Mevkii (Asvan) | 38.870 | 38.970 | Mesopotamia | 3000 | 2800 | 2900 | 3000-2500 | 820 | 447 | 432 | 339 | 500 | 0.033 | 25.000 | 0.000 | 0.000 | 0.00 | 0.00 |  | (96) | (52) |
| Horum Hüyük | 37.100 | 37.867 | Mesopotamia | 3000 | 2800 | 2900 | 3000-2500 | 359 | 522 | 504 | 395 | 584 |  |  |  |  | 5.90 | 0.00 | 1111 |  | (17) |
| Mezraa Hoyük | 36.971 | 37.998 | Mesopotamia | 3000 | 2800 | 2900 | 3000-2500 | 343 | 486 | 468 | 368 | 544 | 8.475 | 60.000 | 0.000 | 0.000 |  |  |  | (97) |  |
| Hassek Höyük | 37.750 | 38.917 | Mesopotamia | 3100 | 2650 | 2875 | 3000-2500 | 530 | 514 | 495 | 389 | 575 | 0.000 | 0.000 | 0.000 | 0.000 |  |  |  | (79) |  |
| Lachish | 31.565 | 34.849 | Southern Levant | 3600 | 2000 | 2800 | 3600-3000 | 267 | 467 | 419 | 233 | 519 | yes | 80.000 | yes | 100.000 | 6.90 | 0.00 | 29 | (98) | (22) |
| Arslantepe | 38.382 | 38.361 | Mesopotamia | 3100 | 2500 | 2800 | 3000-2500 | 902 | 432 | 417 | 327 | 483 | 0.017 | 4.000 | 0.000 | 0.000 | 0.00 | 0.00 | > 119 (some weighed) | (99) | (100) |
| Tell Brak | 36.667 | 41.058 | Mesopotamia | 3100 | 2500 | 2800 | 3000-2500 | 360 | 376 | 363 | 285 | 421 | 0.000 | 0.000 | 0.000 | 0.000 |  |  |  | (46, 57) |  |
| Hirbet ez-Zeraqon | 32.586 | 35.948 | Southern Levant | 3100 | 2500 | 2800 | 3000-2500 | 504 | 392 | 378 | 297 | 438 | 9.641 | 53.000 | 4.915 | 74.000 | 77.80 | 0.10 | 1965 | (101) | (54) |
| Jericho | 31.850 | 35.436 | Southern Levant | 3100 | 2500 | 2800 | 3000-2500 | -209 | 447 | 431 | 338 | 500 | 0.540 | 36.000 | 0.000 | 0.000 | 0.00 | 0.00 | 26 | (4) | (68) |
| Tell Shiukh Fawqani | 36.767 | 38.050 | Mesopotamia | 2900 | 2650 | 2775 | 3000-2500 | 345 | 447 | 431 | 338 | 500 |  |  |  |  | 0.00 | 0.00 | 604 |  | (17) |
| Gre Virike | 36.923 | 38.015 | Mesopotamia | 2900 | 2650 | 2775 | 3000-2500 | 347 | 486 | 468 | 368 | 544 | 1.674 | 100.000 | 0.418 | 33.000 |  |  |  | (102) |  |
| Tepe Yahya | 28.331 | 56.867 | Iran | 3400 | 2100 | 2750 | 3600-3000 | 1513 | 234 | 210 | 117 | 260 | yes | 50.000 | 0.000 | 0.000 |  |  |  | (37) |  |
| Tell Jerablus Tahtani | 36.790 | 38.021 | Mesopotamia | 3100 | 2400 | 2750 | 3000-2500 | 337 | 458 | 442 | 347 | 513 | 44.882 | 67.000 | 0.000 | 0.000 | 0.00 | 0.00 | 2557 | (61, 62) | (63) |
| Tell Fadous-Kfarabida | 34.226 | 35.660 | Northern Levant | 3000 | 2500 | 2750 | 3000-2500 | 21 | 873 | 843 | 661 | 977 | 3.137 | 35.000 | 64.627 | 100.000 | 59.00 | 0.10 | 4384 | (101) | (54) |
| Tell Zagan | 36.549 | 40.752 | Mesopotamia | 2900 | 2500 | 2700 | 3000-2500 | 310 | 357 | 344 | 270 | 399 | 0.000 | 0.000 | 0.000 | 0.000 |  |  |  | (103) |  |
| Tel Yarmouth | 31.708 | 34.975 | Southern Levant | 2900 | 2500 | 2700 | 3000-2500 | 399 | 546 | 526 | 413 | 610 | 4.892 | 38.000 | 74.983 | 75.000 | 67.30 | 0.00 | 446 | (93) | (93) |
| Tell Taannach | 32.522 | 35.220 | Southern Levant | 2900 | 2500 | 2700 | 3000-2500 | 168 | 651 | 628 | 493 | 728 | 0.000 | 0.000 | 0.000 | 0.000 |  |  |  | (75) |  |
| Hajji Ibrahim | 36.340 | 38.210 | Mesopotamia | 3100 | 2300 | 2700 | 3000-2500 | 332 | 372 | 358 | 281 | 416 | 0.000 | 0.000 | 0.000 | 0.000 |  |  |  | (104) |  |
| Numeira | 31.130 | 35.529 | Southern Levant | 2900 | 2500 | 2700 | 3000-2500 | -280 | 123 | 119 | 93 | 138 | yes |  | yes |  |  |  |  | (105) |  |
| Tell Ashara_Terqa | 34.903 | 40.530 | Mesopotamia | 2900 | 2450 | 2675 | 3000-2500 | 196 | 151 | 146 | 114 | 169 | 0.320 | 17.000 | 0.000 | 0.000 |  |  |  | (106) |  |
| Megiddo | 32.585 | 35.185 | Southern Levant | 2900 | 2450 | 2675 | 3000-2500 | 162 | 644 | 622 | 488 | 721 | yes |  | 3.636 | 29.000 | 42.00 | 0.00 | 81 | (73) | (93) |
| Bab'edh Dhra | 31.253 | 35.533 | Southern Levant | 2900 | 2450 | 2675 | 3000-2500 | -247 | 194 | 188 | 147 | 217 | 8.136 | 53.000 | 0.169 | 7.000 |  |  |  | (87) |  |
| Beth Shean | 32.504 | 35.503 | Southern Levant | 2900 | 2450 | 2675 | 3000-2500 | -120 | 508 | 490 | 397 | 569 | 20.000 | 75.000 | 4.000 | 25.000 |  |  |  | (89) |  |
| Tell al-Raqa'i | 36.430 | 40.870 | Mesopotamia | 2900 | 2400 | 2650 | 3000-2500 | 291 | 346 | 334 | 262 | 387 | 0.170 | 5.000 | 0.000 | 0.000 |  |  |  | (107) |  |
| Sidon | 33.560 | 35.371 | Southern Levant | 2800 | 2500 | 2650 | 3000-2500 | 20 | 826 | 797 | 625 | 924 | 0.000 | 0.000 | 0.000 | 0.000 | yes |  |  | (108) | (109, 110) |
| Kish | 32.550 | 44.650 | Mesopotamia | 2900 | 2350 | 2625 | 3000-2500 | 35 | 126 | 121 | 95 | 140 | 0.000 | 0.000 | 0.000 | 0.000 |  |  |  | (111) |  |
| Shahr-i Sokhta | 31.000 | 61.000 | Iran | 3200 | 2000 | 2600 | 3000-2500 | 487 | 26 | 25 | 20 | 29 | 0.000 | 0.000 | 0.000 | 0.000 | 0.00 | 0.00 | 1318 | (37) | (37) |
| Tappeh Gijlar | 37.930 | 45.300 | Iran | 3200 | 2000 | 2600 | 3000-2500 | 1267 | 345 | 332 | 261 | 385 | 0.000 | 0.000 | 0.000 | 0.000 |  |  |  | (44) |  |
| Abu Salabikh | 32.252 | 45.053 | Mesopotamia | 2900 | 2300 | 2600 | 3000-2500 | 26 | 130 | 126 | 99 | 146 | 0.000 | 0.000 | 0.000 | 0.000 |  |  |  | (112) |  |
| Tell Atij | 36.430 | 40.850 | Mesopotamia | 2900 | 2300 | 2600 | 3000-2500 | 293 | 345 | 333 | 261 | 386 | 0.234 | 12.000 | 0.000 | 0.000 | 0.00 | 0.00 | 128 | (113) | (114) |
| Konar Sandal | 28.459 | 57.782 | Iran | 3100 | 2000 | 2550 | 3000-2500 | 577 | 51 | 49 | 38 | 57 | 26.230 | 33.000 | 0.000 | 0.000 | 3.00 | 0.10 | 1453 | (115) | (115) |
| Tell es-Sweyhat | 36.274 | 38.254 | Mesopotamia | 3100 | 2000 | 2550 | 3000-2500 | 334 | 365 | 352 | 276 | 408 | 0.000 | 0.000 | 0.000 | 0.000 |  |  |  | (116) |  |
| Tell Kerma | 36.448 | 40.837 | Mesopotamia | 3100 | 2000 | 2550 | 3000-2500 | 290 | 345 | 333 | 261 | 386 | 0.000 | 0.000 | 0.000 | 0.000 |  |  |  | (113) |  |
| Titris Höyük | 37.480 | 38.620 | Mesopotamia | 3100 | 2000 | 2550 | 3000-2500 | 530 | 556 | 536 | 421 | 622 | 0.000 | 0.000 | 0.000 | 0.000 |  |  |  |  |  |
| Tell Nebi Mend (Kadesh) | 34.556 | 36.519 | Northern Levant | 3100 | 2000 | 2550 | 3000-2500 | 511 | 512 | 494 | 387 | 572 | 2.540 | 67.000 | 0.000 | 0.000 |  |  |  | (117) |  |
| Tell Bderi | 36.388 | 40.814 | Mesopotamia | 2750 | 2300 | 2525 | 3000-2500 | 297 | 339 | 327 | 256 | 379 | 0.017 | 9.000 | 0.000 | 0.000 | 0.06 | 0.30 | 1801 | (107, 118) | (119) |
| Korucutepe | 38.467 | 39.550 | Mesopotamia | 2800 | 2200 | 2500 | 3000-2500 | 820 | 543 | 524 | 411 | 607 | 0.000 | 0.000 | 0.000 | 0.000 | 0.00 | 0.00 | 68 | (45) | (45) |
| Tell Jerablus Tahtani | 36.790 | 38.021 | Mesopotamia | 2650 | 2300 | 2475 | 2500-2000 | 337 | 458 | 398 | 349 | 459 | yes |  | 0.000 | 0.000 | 0.60 | 0.50 | 1589 | (120) | (63) |
| Kurban Höyük | 37.476 | 38.429 | Mesopotamia | 2900 | 2000 | 2450 | 3000-2500 | 530 | 570 | 550 | 432 | 638 | yes; 0 |  | 0.000 | 0.000 | 0.00 | 11.10 | 234 | (121, 122) | (123) |
| Tell el'Abd | 36.250 | 38.180 | Mesopotamia | 2900 | 2000 | 2450 | 3000-2500 | 302 | 354 | 342 | 268 | 396 | 0.000 | 0.000 | 0.000 | 0.000 | 0.00 | 0.00 | 1284 | (124) | (125) |
| Tell Hammam et-Turkman | 36.483 | 39.057 | Mesopotamia | 2900 | 2000 | 2450 | 3000-2500 | 339 | 365 | 352 | 276 | 408 | 0.581 | 50.000 | 0.000 | 0.000 |  |  |  | (49) |  |
| Ebla | 35.798 | 36.798 | Northern Levant | 2550 | 2300 | 2425 | 2500-2000 | 428 | 359 | 312 | 273 | 360 | 1.529 | 60.000 | 29.664 | 100.000 | 6.03 | 0.00 | 464 | (126) | (127, 128) |
| Asvan Kale (Asvan) | 38.900 | 38.950 | Mesopotamia | 2800 | 2000 | 2400 | 3000-2500 | 820 | 448 | 433 | 339 | 501 | 0.622 | 42.000 | 0.000 | 0.000 | 0.00 | 0.00 | n.a. | (96) | (52) |
| Tell Shiukh Fawqani | 36.767 | 38.050 | Mesopotamia | 2450 | 2300 | 2375 | 2500-2000 | 345 | 447 | 388 | 340 | 448 | 0.000 | 0.000 | 0.000 | 0.000 | 0.00 | 0.00 | 394 | (95) | (17) |
| Gre Virike | 36.923 | 38.015 | Mesopotamia | 2650 | 2100 | 2375 | 2500-2000 | 347 | 486 | 422 | 370 | 487 | 42.308 | 50.000 | 7.692 | 17.000 |  |  |  | (102) |  |
| Tell Abu en-Ni'aj | 32.426 | 35.566 | Southern Levant | 2500 | 2250 | 2375 | 2500-2000 | -252 | 485 | 421 | 369 | 486 | 3.496 | 23.000 | 0.624 | 10.000 |  |  |  | (129) |  |
| Tell al-Rawda | 35.181 | 37.633 | Northern Levant | 2500 | 2200 | 2350 | 2500-2000 | 517 | 286 | 248 | 218 | 287 | 0.000 | 0.000 | 0.093 | 14.000 |  |  |  | (130) |  |
| Tell Qara Quzaq | 36.633 | 38.217 | Mesopotamia | 2650 | 2000 | 2325 | 2500-2000 | 325 | 424 | 368 | 323 | 425 | 2.062 | 50.000 | 0.000 | 0.000 | 0.00 | 0.00 | 496 | (131) | (54) |
| Mezraa Hoyük | 36.971 | 37.998 | Mesopotamia | 2600 | 2000 | 2300 | 2500-2000 | 343 | 486 | 422 | 370 | 487 | 6.452 | 100.000 | 0.000 | 0.000 |  |  |  | (97) |  |
| Tell Mishrifeh | 34.834 | 36.866 | Northern Levant | 2400 | 2200 | 2300 | 2500-2000 | 500 | 313 | 271 | 238 | 313 | 2.425 | 33.000 | 0.000 | 0.000 | 12.30 | 3.30 | 244 | (132, 133) | (134) |
| Tell Tweini | 35.372 | 35.936 | Northern Levant | 2600 | 2000 | 2300 | 2500-2000 | 26 | 756 | 656 | 575 | 758 | 5.941 |  | 28.713 |  |  |  |  | (135) |  |
| Emar | 35.987 | 38.111 | Mesopotamia | 2450 | 2100 | 2275 | 2500-2000 | 305 | 322 | 280 | 245 | 323 | 0.215 | 15.000 | 0.107 | 8.000 | 0.02 | 0.00 | 18768 | (136) | (137, 138) |
| Tell Leilan | 36.957 | 41.505 | Mesopotamia | 2350 | 2200 | 2275 | 2500-2000 | 388 | 426 | 369 | 324 | 426 | 0.000 | 0.000 | 0.000 | 0.000 | 0.00 | 0.20 | 407 | (139) | (140) |
| Tell Mozan | 37.057 | 40.997 | Mesopotamia | 2550 | 2000 | 2275 | 2500-2000 | 471 | 434 | 376 | 330 | 435 | 0.128 | 33.000 | 0.000 | 0.000 | 0.00 | 0.00 | 8827 | (141) | (142) |
| Tell Taya | 36.333 | 42.494 | Mesopotamia | 2350 | 2200 | 2275 | 2500-2000 | 369 | 366 | 317 | 278 | 366 | 8.696 | 5.000 | 19.565 | 5.000 |  |  |  | (143) |  |
| Tell Nebi Mend (Kadesh) | 34.556 | 36.519 | Northern Levant | 2550 | 2000 | 2275 | 2500-2000 | 511 | 512 | 444 | 389 | 513 | 7.423 | 71.000 | 0.000 | 0.000 |  |  |  | (117, 144) |  |
| Ur | 30.962 | 46.105 | Mesopotamia | 2500 | 2000 | 2250 | 2500-2000 | 14 | 104 | 101 | 79 | 117 | 0.000 | 0.000 | 0.000 | 0.000 |  |  |  | (145) |  |
| Jericho | 31.850 | 35.436 | Southern Levant | 2500 | 2000 | 2250 | 2500-2000 | -209 | 447 | 388 | 340 | 447 | 0.000 | 0.000 | 0.000 | 0.000 | 0.00 | 0.60 | 166 | (4) | (68) |
| Tell el-Hayyat | 32.421 | 35.577 | Southern Levant | 2500 | 2000 | 2250 | 2500-2000 | -239 | 485 | 421 | 369 | 486 | 0.000 | 0.000 | yes |  |  |  |  | (146) |  |
| Bab'edh Dhra | 31.253 | 35.533 | Southern Levant | 2500 | 2000 | 2250 | 2500-2000 | -247 | 194 | 168 | 147 | 194 | 4.412 | 56.000 | 9.314 | 33.000 |  |  |  | (87) |  |
| Tell Selenkahiye | 36.100 | 38.050 | Mesopotamia | 2450 | 2000 | 2225 | 2500-2000 | 307 | 334 | 289 | 254 | 334 | 0.000 | 0.000 | 0.000 | 0.000 | 0.00 | 0.00 | 1101.65 gr | (116) | (116) |
| Tell Brak | 36.667 | 41.058 | Mesopotamia | 2500 | 1950 | 2225 | 2500-2000 | 360 | 376 | 327 | 286 | 377 | 0.029 | 18.000 | 0.000 | 0.000 |  |  |  | (46, 57) |  |
| Tell Qarqur | 35.742 | 36.331 | Northern Levant | 2450 | 2000 | 2225 | 2500-2000 | 193 | 481 | 417 | 366 | 481 | 0.091 | 22.000 | 0.003 | 2.000 |  |  |  | (147) |  |
| Horum Hüyük | 37.100 | 37.867 | Mesopotamia | 2300 | 2100 | 2200 | 2500-2000 | 359 | 522 | 453 | 397 | 523 |  |  |  |  | 0.20 | 0.30 | 2604 |  | (17) |
| Tell Afis | 35.905 | 36.799 | Northern Levant | 2300 | 2100 | 2200 | 2500-2000 | 360 | 356 | 309 | 271 | 357 | 0.275 | 12.000 | 8.791 | 62.000 |  |  |  | (42) |  |
| Imamoglu | 38.483 | 38.450 | Mesopotamia | 2300 | 2000 | 2150 | 2500-2000 | 675 | 416 | 361 | 317 | 417 | 0.000 | 0.000 | 0.000 | 0.000 |  |  |  | (148) |  |
| Umm el-Marra | 36.134 | 37.693 | Northern Levant | 2300 | 2000 | 2150 | 2500-2000 | 333 | 343 | 298 | 261 | 344 | 1.136 | 25.000 | 0.000 | 0.000 |  |  |  | (149) |  |
| Tell Fadous-Kfarabida | 34.226 | 35.660 | Northern Levant | 2250 | 2000 | 2125 | 2500-2000 | 21 | 873 | 758 | 664 | 875 | 12.500 | 25.000 | 62.500 | 100.000 |  |  |  | (101) |  |
| Malyan | 30.050 | 52.420 | Iran | 2400 | 1800 | 2100 | 2500-2000 | 1618 | 254 | 221 | 193 | 255 | 0.000 | 0.000 | 0.000 | 0.000 | 0.00 | 1.20 | 2019 | (92) | (92) |
| Tepe Yahya | 28.331 | 56.867 | Iran | 2400 | 1800 | 2100 | 2500-2000 | 1513 | 234 | 203 | 178 | 235 | 0.000 | 0.000 | 0.000 | 0.000 |  |  |  | (37) |  |
| Tell Arbid | 36.872 | 41.022 | Mesopotamia | 2100 | 2000 | 2050 | 2500-2000 | 396 | 410 | 356 | 312 | 411 | 0.000 | 0.000 | 0.000 | 0.000 |  |  |  | (150) |  |
| Tell Ashara_Terqa | 34.903 | 40.530 | Mesopotamia | 2200 | 1900 | 2050 | 2500-2000 | 196 | 151 | 131 | 115 | 151 | 0.000 | 0.000 | 0.000 | 0.000 |  |  |  | (106) |  |
| Tell Qurtass | 35.742 | 36.331 | Mesopotamia | 2100 | 1950 | 2025 | 2500-2000 | 191 | 481 | 417 | 366 | 481 | 0.000 | 0.000 | 0.000 | 0.000 |  |  |  | (41) |  |
| Tepe Hissar | 36.150 | 54.070 | Iran | 2200 | 1800 | 2000 | 2000-1500 | 1621 | 205 | 170 | 158 | 184 | 0.000 | 0.000 | 0.000 | 0.000 |  |  |  | (83) |  |
| Tell Burak | 33.482 | 35.322 | Southern Levant | 2000 | 1800 | 1900 | 2000-1500 | 8 | 789 | 654 | 609 | 707 | 12.981 | 43.000 | 9.135 | 53.000 | 37.60 | 0.00 | 1148 | (151) | (152) |
| Tell el Ifshar | 32.372 | 34.908 | Southern Levant | 2000 | 1800 | 1900 | 2000-1500 | 20 | 670 | 555 | 517 | 601 | 0.000 | 0.000 | 0.000 | 0.000 |  |  |  | (153) |  |
| Zahrat adh-Dhra 1 | 31.257 | 35.566 | Southern Levant | 2000 | 1800 | 1900 | 2000-1500 | -146 | 198 | 164 | 153 | 178 | 15.281 |  | 0.000 | 0.000 |  |  |  | (154) |  |
| Tell Mozan | 37.057 | 40.997 | Mesopotamia | 2000 | 1700 | 1850 | 2000-1500 | 471 | 434 | 360 | 335 | 389 | 0.902 | 6.000 | 0.000 | 0.000 | 30.03 | 1.22 | 1152 | (141) | (142) |
| Tell Arbid | 36.872 | 41.022 | Mesopotamia | 2000 | 1700 | 1850 | 2000-1500 | 396 | 410 | 340 | 317 | 368 | 0.000 | 0.000 | 0.000 | 0.000 |  |  |  | (150) |  |
| Tepe Farukhabad | 32.587 | 47.224 | Iran | 2350 | 1300 | 1825 | 2000-1500 | 140 | 260 | 215 | 201 | 233 | 0.000 | 0.000 | 0.000 | 0.000 |  |  |  | (27) |  |
| Emar | 35.987 | 38.111 | Mesopotamia | 2000 | 1600 | 1800 | 2000-1500 | 305 | 322 | 267 | 249 | 289 | 3.191 | 25.000 | 0.000 | 0.000 | 0.00 | 0.00 | 1541 | (136) | (137, 138, 155) |
| Umm el-Marra | 36.134 | 37.693 | Northern Levant | 2000 | 1600 | 1800 | 2000-1500 | 333 | 343 | 284 | 265 | 307 | 3.252 | 43.000 | 0.000 | 0.000 |  |  |  | (156) |  |
| Ebla | 35.798 | 36.798 | Northern Levant | 2000 | 1600 | 1800 | 2000-1500 | 428 | 359 | 298 | 278 | 322 | 2.545 | 71.000 | 13.232 | 57.000 | 40.60 | 0.00 | 1234 | (126) | (157) |
| Kinet Höyük | 36.854 | 36.157 | Northern Levant | 2000 | 1600 | 1800 | 2000-1500 | 20 | 695 | 576 | 537 | 623 | 0.000 | 0.000 | 0.000 | 0.000 | 0.00 | 0.00 | 3100 | (158, 159) | (54) |
| Baalbek | 34.017 | 36.200 | Northern Levant | 2000 | 1600 | 1800 | 2000-1500 | 1144 | 606 | 502 | 468 | 543 |  |  |  |  | 0.10 | 0.00 | 871 |  | (160) |
| Jebel Mousa | 34.072 | 35.773 | Northern Levant | 2000 | 1600 | 1800 | 2000-1500 | 851 | 895 | 741 | 691 | 802 |  |  |  |  | 0.00 | 0.00 | 417 |  | (54) |
| Tell Afis | 35.905 | 36.799 | Northern Levant | 2000 | 1600 | 1800 | 2000-1500 | 360 | 356 | 295 | 275 | 320 | 0.000 | 0.000 | 0.000 | 0.000 |  |  |  | (42) |  |
| Tell Mishrifeh | 34.834 | 36.866 | Northern Levant | 2000 | 1600 | 1800 | 2000-1500 | 500 | 313 | 259 | 242 | 280 | 0.135 | 25.000 | 0.000 | 0.000 | 1.16 | 2.80 | 1118 | (134) | (134) |
| Tell Fadous-Kfarabida | 34.226 | 35.660 | Northern Levant | 2000 | 1600 | 1800 | 2000-1500 | 21 | 873 | 724 | 675 | 783 | 10.112 | 75.000 | 28.090 | 100.000 |  |  |  | (101) |  |
| Sidon | 33.560 | 35.371 | Southern Levant | 2000 | 1600 | 1800 | 2000-1500 | 20 | 826 | 684 | 638 | 741 | 2.820 | 47.000 | 9.492 | 82.000 |  |  |  | (161) |  |
| Tell Nebi Mend (Kadesh) | 34.556 | 36.519 | Northern Levant | 2000 | 1600 | 1800 | 2000-1500 | 511 | 512 | 424 | 395 | 459 | 36.585 | 100.000 | 0.000 | 0.000 |  |  |  | (117) |  |
| Afula | 32.611 | 35.290 | Southern Levant | 2000 | 1600 | 1800 | 2000-1500 | 65 | 613 | 508 | 473 | 549 | 0.000 | 0.000 | 0.000 | 0.000 |  |  |  | (59) |  |
| Tell Tweini | 35.372 | 35.936 | Northern Levant | 2000 | 1600 | 1800 | 2000-1500 | 26 | 756 | 627 | 584 | 678 | 18.904 | 83.000 | 11.732 | 89.000 |  |  |  | (135) |  |
| Tell Hadidi | 36.264 | 38.151 | Mesopotamia | 2000 | 1550 | 1775 | 2000-1500 | 313 | 365 | 302 | 282 | 327 | 0.000 | 0.000 | 0.000 | 0.000 | 0.00 | 0.00 | 956 gr | (116) | (116) |
| Tell Hammam et-Turkman | 36.483 | 39.057 | Mesopotamia | 2000 | 1550 | 1775 | 2000-1500 | 339 | 365 | 303 | 282 | 327 | 0.000 | 0.000 | 0.000 | 0.000 |  |  |  | (49) |  |
| Korucutepe | 38.467 | 39.550 | Mesopotamia | 2000 | 1550 | 1775 | 2000-1500 | 820 | 543 | 450 | 419 | 487 | 0.000 | 0.000 | 0.000 | 0.000 |  |  |  | (45) |  |
| Mezraa Hoyük | 36.971 | 37.998 | Mesopotamia | 2000 | 1550 | 1775 | 2000-1500 | 343 | 486 | 403 | 376 | 436 | 61.111 | 50.000 | 0.000 | 0.000 |  |  |  | (97, 102) |  |
| Tell Brak | 36.667 | 41.058 | Mesopotamia | 2000 | 1550 | 1775 | 2000-1500 | 360 | 376 | 312 | 291 | 338 | 0.008 | 3.000 | 0.000 | 0.000 |  |  |  | (57) |  |
| Tell Qara Quzaq | 36.633 | 38.217 | Mesopotamia | 2000 | 1550 | 1775 | 2000-1500 | 325 | 424 | 352 | 328 | 380 | 2.047 | 43.000 | 0.000 | 0.000 |  |  |  | (131) |  |
| Jericho | 31.850 | 35.436 | Southern Levant | 2000 | 1550 | 1775 | 2000-1500 | -209 | 447 | 370 | 345 | 401 | 0.003 | 15.000 | 0.000 | 0.000 | 0.00 | 0.00 | 26 | (4) | (68) |
| Megiddo | 32.585 | 35.185 | Southern Levant | 2000 | 1550 | 1775 | 2000-1500 | 162 | 644 | 534 | 498 | 578 | 0.000 | 0.000 | 3.721 | 50.000 | 64.70 | 0.00 | 99 | (73) | (74) |
| Tel Yoqneam/Tell Qemun/Jokneam | 32.665 | 35.109 | Southern Levant | 2000 | 1550 | 1775 | 2000-1500 | 99 | 675 | 559 | 521 | 605 | 0.000 | 0.000 | yes |  |  |  |  | (75) |  |
| Tell Aphek | 32.105 | 34.931 | Southern Levant | 2000 | 1550 | 1775 | 2000-1500 | 36 | 606 | 503 | 468 | 544 | 0.000 | 0.000 | yes |  | 38.60 | 0.00 | 70 | (75) | (22) |
| Tell el-Hayyat | 32.421 | 35.577 | Southern Levant | 2000 | 1550 | 1775 | 2000-1500 | -239 | 485 | 402 | 375 | 435 | 0.766 | 23.000 | 2.710 | 30.000 |  |  |  | (146) |  |
| Tell Gerisa | 32.092 | 34.808 | Southern Levant | 2000 | 1550 | 1775 | 2000-1500 | 25 | 578 | 479 | 447 | 519 | 0.000 | 0.000 | yes |  | yes |  |  | (75) | (22) |
| Tell Taannach | 32.522 | 35.220 | Southern Levant | 2000 | 1550 | 1775 | 2000-1500 | 168 | 651 | 539 | 503 | 584 | 0.000 | 0.000 | yes |  | yes |  |  | (75) | (22) |
| er-Rukeis | 32.340 | 36.571 | Southern Levant | 2000 | 1550 | 1775 | 2000-1500 | 921 | 174 | 144 | 134 | 156 | 0.000 | 0.000 | 0.000 | 0.000 |  |  |  | (31) |  |
| Tell ed-Der | 33.100 | 44.300 | Mesopotamia | 1900 | 1600 | 1750 | 2000-1500 | 40 | 144 | 119 | 111 | 129 | 0.000 | 0.000 | 0.000 | 0.000 |  |  |  | (162) |  |
| Chagar Bazar | 36.876 | 40.898 | Mesopotamia | 2000 | 1500 | 1750 | 2000-1500 | 393 | 407 | 338 | 315 | 365 |  |  |  |  | 0.00 | 0.00 | 183 |  | (163) |
| Kenan Tepe | 37.831 | 40.813 | Mesopotamia | 2000 | 1500 | 1750 | 2000-1500 | 564 | 490 | 406 | 378 | 439 | 0.000 | 0.000 | 0.000 | 0.000 |  |  |  | (164) |  |
| Tell Khamîs | 36.718 | 38.186 | Mesopotamia | 2000 | 1450 | 1725 | 2000-1500 | 26 | 429 | 355 | 331 | 384 | yes |  | 0.000 | 0.000 |  |  |  | (165) |  |
| City of David_Jerusalem | 31.772 | 35.235 | Southern Levant | 1800 | 1600 | 1700 | 2000-1500 | 703 | 620 | 514 | 479 | 556 |  |  | yes |  |  |  |  | (69) |  |
| Manahat | 31.752 | 35.183 | Southern Levant | 1800 | 1600 | 1700 | 2000-1500 | 717 | 613 | 508 | 473 | 550 | 33.557 | 44.000 | 25.168 | 56.000 |  |  |  | (166) |  |
| Lachish | 31.565 | 34.849 | Southern Levant | 1800 | 1550 | 1675 | 2000-1500 | 267 | 467 | 387 | 361 | 419 | 8.333 | 16.000 | 25.000 | 55.000 | 63.00 | 0.00 | 1496 | (167) | (22, 54) |
| Beth Shean | 32.504 | 35.503 | Southern Levant | 1750 | 1550 | 1650 | 2000-1500 | -120 | 508 | 441 | 387 | 509 | 5.738 | 50.000 | 25.137 | 70.000 | 80.00 | 0.00 | 30 | (168) | (169) |
| Zincirli | 37.103 | 36.678 | Northern Levant | 1700 | 1600 | 1650 | 2000-1500 | 501 | 604 | 500 | 466 | 541 | 0.080 | 31.000 | 0.020 | 13.000 | 3.30 | 0.00 | 3326 | (170) | (170) |
| Shiloh | 32.056 | 35.290 | Southern Levant | 1750 | 1550 | 1650 | 2000-1500 | 712 | 625 | 518 | 483 | 561 | 13.595 |  | 32.628 |  |  |  |  | (171, 172) |  |
| Timnah. Tel Batash | 31.784 | 34.911 | Southern Levant | 1750 | 1550 | 1650 | 2000-1500 | 129 | 540 | 448 | 417 | 484 |  |  | yes |  | 34.80 | 0.00 | 23 | (173) | (22) |
| Tell Atchana | 36.238 | 36.384 | Northern Levant | 2000 | 1200 | 1600 | 2000-1500 | 96 | 522 | 433 | 403 | 468 | 5.000 | 50.000 | 5.000 | 25.000 |  |  |  | (174) |  |
| Tell Bazi | 36.426 | 38.275 | Mesopotamia | 1800 | 1350 | 1575 | 1500-1200 | 364 | 387 | 335 | 324 | 357 |  |  |  |  | 0.00 | 0.06 | 1688 |  | (54) |
| Tell Abu al-Kharaz | 32.399 | 35.595 | Southern Levant | 1600 | 1400 | 1500 | 1500-1200 | -133 | 479 | 415 | 402 | 443 | 0.000 | 0.000 | 0.000 | 0.000 |  |  |  | (81) |  |
| Tell Burak | 33.482 | 35.322 | Southern Levant | 1600 | 1400 | 1500 | 1500-1200 | 8 | 789 | 683 | 661 | 729 | 6.504 | 57.000 | 7.317 | 71.000 | 14.50 | 0.00 | 179 | (151) | (152) |
| Tell Hadidi | 36.264 | 38.151 | Mesopotamia | 1550 | 1400 | 1475 | 1500-1200 | 313 | 365 | 316 | 306 | 337 | 0.000 | 0.000 | 0.000 | 0.000 | 0.00 | 0.00 | 3327.31 gr | (116) | (116) |
| Tell Bderi | 36.388 | 40.814 | Mesopotamia | 1550 | 1350 | 1450 | 1500-1200 | 297 | 339 | 293 | 284 | 313 | 12.500 | 33.000 | 0.000 | 0.000 |  |  |  | (118) |  |
| Tell el Ifshar | 32.372 | 34.908 | Southern Levant | 1600 | 1300 | 1450 | 1500-1200 | 20 | 670 | 580 | 562 | 619 | 0.000 | 0.000 | 0.000 | 0.000 |  |  |  | (153) |  |
| Emar | 35.987 | 38.111 | Mesopotamia | 1600 | 1200 | 1400 | 1500-1200 | 305 | 322 | 279 | 270 | 298 | 12.690 | 21.000 | 1.015 | 13.000 | 0.00 | 0.01 | 6674 | (136) | (137, 138) |
| Korucutepe | 38.467 | 39.550 | Mesopotamia | 1600 | 1200 | 1400 | 1500-1200 | 820 | 543 | 470 | 455 | 502 | 0.000 | 0.000 | 0.000 | 0.000 |  |  |  | (45) |  |
| Asvan Kale (Asvan) | 38.900 | 38.950 | Mesopotamia | 1600 | 1200 | 1400 | 1500-1200 | 820 | 448 | 388 | 376 | 414 | 0.377 | 50.000 | 0.000 | 0.000 |  |  |  | (96) |  |
| Tell Munbāqa | 36.217 | 38.130 | Mesopotamia | 1600 | 1200 | 1400 | 1500-1200 | 318 | 355 | 308 | 298 | 328 | 0.000 | 0.000 | 0.021 | 9.000 |  |  |  | (175) |  |
| Tilbeshar | 36.873 | 37.559 | Mesopotamia | 1600 | 1200 | 1400 | 1500-1200 | 604 | 535 | 463 | 448 | 494 | 30.968 |  | 1.935 |  |  |  |  | (31) |  |
| Kinet Höyük | 36.854 | 36.157 | Northern Levant | 1600 | 1200 | 1400 | 1500-1200 | 20 | 695 | 602 | 583 | 642 | 4.337 | 29.000 | 0.241 | 2.703 | 49.82 | 0.71 | 283 | (158, 159) | (54) |
| Tell Mishrifeh | 34.834 | 36.866 | Northern Levant | 1600 | 1200 | 1400 | 1500-1200 | 500 | 313 | 271 | 262 | 289 | 72.500 | 13.000 | 8.438 | 13.000 | 14.10 | 1.30 | 6292 | (133) | (176) |
| Umm el-Marra | 36.134 | 37.693 | Northern Levant | 1600 | 1200 | 1400 | 1500-1200 | 333 | 343 | 297 | 287 | 317 | 0.935 | 21.000 | 0.000 | 0.000 |  |  |  | (156) |  |
| Megiddo | 32.585 | 35.185 | Southern Levant | 1600 | 1200 | 1400 | 1500-1200 | 162 | 644 | 558 | 540 | 595 | 0.000 | 0.000 | 86.415 | 67.000 | 55.90 | 0.50 | 195 | (73, 177) | (74) |
| Sidon | 33.560 | 35.371 | Southern Levant | 1600 | 1200 | 1400 | 1500-1200 | 20 | 826 | 715 | 692 | 763 | 0.704 | 17.000 | 59.859 | 100.000 |  |  |  | (178) |  |
| Tel Michal | 32.161 | 34.797 | Southern Levant | 1600 | 1200 | 1400 | 1500-1200 | 14 | 608 | 526 | 510 | 562 |  |  | yes |  |  |  |  | (179) |  |
| Tell Gerisa | 32.092 | 34.808 | Southern Levant | 1600 | 1200 | 1400 | 1500-1200 | 25 | 578 | 501 | 485 | 534 |  |  |  |  | 42.30 | 0.00 | 26 |  | (22) |
| Tell Sera | 31.391 | 34.681 | Southern Levant | 1600 | 1200 | 1400 | 1500-1200 | 160 | 351 | 304 | 294 | 324 | 0.000 | 0.000 | yes |  | 32.70 | 0.00 | 245 | (75) | (22) |
| Tell Taannach | 32.522 | 35.220 | Southern Levant | 1600 | 1200 | 1400 | 1500-1200 | 168 | 651 | 564 | 546 | 602 | yes |  | 0.000 | 0.000 | 65.00 | 0.00 | 20 | (75) | (22) |
| Kamid el-Loz | 33.624 | 35.821 | Southern Levant | 1600 | 1200 | 1400 | 1500-1200 | 934 | 710 | 615 | 595 | 656 | 0.302 | 100.000 | 0.000 | 0.000 |  |  |  | (180, 181) |  |
| Pella | 32.450 | 35.613 | Southern Levant | 1600 | 1200 | 1400 | 1500-1200 | -37 | 482 | 418 | 405 | 446 | 6.250 | 20.000 | 0.000 | 0.000 |  |  |  | (29) |  |
| Tell Halif | 31.383 | 34.867 | Southern Levant | 1600 | 1200 | 1400 | 1500-1200 | 483 | 391 | 338 | 328 | 361 | 0.000 | 0.000 | 0.000 | 0.000 |  |  |  | (40) |  |
| Tell Nebi Mend (Kadesh) | 34.556 | 36.519 | Northern Levant | 1600 | 1200 | 1400 | 1500-1200 | 511 | 512 | 443 | 429 | 473 | 3.670 | 100.000 | 0.000 | 0.000 |  |  |  | (117, 144) |  |
| Tell Tweini | 35.372 | 35.936 | Northern Levant | 1600 | 1200 | 1400 | 1500-1200 | 26 | 756 | 655 | 634 | 699 | 14.132 | 60.000 | 7.726 | 83.000 |  |  |  | (135) |  |
| Tell Hwes | 36.548 | 40.757 | Mesopotamia | 1550 | 1200 | 1375 | 1500-1200 | 309 | 357 | 309 | 299 | 330 | 0.000 | 0.000 | 0.000 | 0.000 |  |  |  | (103) |  |
| Tell Atchana | 36.238 | 36.384 | Northern Levant | 1550 | 1200 | 1375 | 1500-1200 | 96 | 522 | 452 | 438 | 483 | 13.682 | 43.000 | 0.746 | 13.000 | 6.10 | 0.20 | 984 | (174) | (155) |
| Beth Shean | 32.504 | 35.503 | Southern Levant | 1550 | 1200 | 1375 | 1500-1200 | -120 | 508 | 440 | 426 | 470 | 0.009 | 66.000 | 0.030 | 33.000 | 55.30 | 0.00 | 38 | (168) | (182) |
| Tel Yoqneam_Tell Qemun_Jokneam | 32.665 | 35.109 | Southern Levant | 1550 | 1200 | 1375 | 1500-1200 | 99 | 675 | 584 | 566 | 623 | yes |  | yes |  |  |  |  | (75) |  |
| Tell Shiukh Fawqani | 36.767 | 38.050 | Mesopotamia | 1400 | 1200 | 1300 | 1500-1200 | 345 | 447 | 387 | 375 | 413 | 0.000 | 0.000 | 0.000 | 0.000 | 0.00 | 0.00 | 1132 | (95) | (17) |
| Tell Afis | 35.905 | 36.799 | Northern Levant | 1400 | 1200 | 1300 | 1500-1200 | 360 | 356 | 309 | 299 | 329 | 3.058 | 31.000 | 3.976 | 23.000 |  |  |  | (42) |  |
| Tell Aphek | 32.105 | 34.931 | Southern Levant | 1400 | 1200 | 1300 | 1500-1200 | 36 | 606 | 525 | 509 | 560 | 95.044 | 100.000 | 0.055 | 80.000 | 17.85 | 0.00 | 84 | (183) | (22, 184) |
| Tall al-'Umayri | 31.869 | 35.888 | Southern Levant | 1400 | 1200 | 1300 | 1500-1200 | 904 | 307 | 265 | 257 | 283 | 1.654 | 50.000 | 0.000 | 0.000 |  |  |  | (185) |  |
| Deir'Alla | 32.196 | 35.621 | Southern Levant | 1300 | 1200 | 1250 | 1500-1200 | -219 | 438 | 379 | 367 | 405 | 0.000 | 0.000 | 0.028 | 13.000 | 32.80 |  |  | (186) | (25) |
| Lachish | 31.565 | 34.849 | Southern Levant | 1400 | 1100 | 1250 | 1500-1200 | 267 | 467 | 404 | 392 | 431 | 0.000 | 0.000 | 86.154 |  | 27.73 | 0.00 | 1976 | (187) | (22, 54) |
| Tell el-Fukhar | 32.589 | 35.953 | Southern Levant | 1300 | 1200 | 1250 | 1500-1200 | 434 | 392 | 339 | 329 | 362 | 0.000 | 0.000 | 0.000 | 0.000 |  |  |  | (94) |  |
| Ashdod | 31.756 | 34.658 | Southern Levant | 1300 | 1200 | 1250 | 1500-1200 | 35 | 501 | 433 | 420 | 463 | 99.977 | 75.000 | 0.000 | 0.000 |  |  |  | (188) |  |
| Deir el-Balah | 31.417 | 34.351 | Southern Levant | 1300 | 1200 | 1250 | 1500-1200 | 23 | 314 | 272 | 263 | 290 | 0.000 | 0.000 | 0.000 | 0.000 |  |  |  | (189) |  |
| Qubur al-Walaydah | 31.413 | 34.534 | Southern Levant | 1300 | 1200 | 1250 | 1500-1200 | 79 | 340 | 294 | 285 | 314 | 5.357 | 11.000 | 0.000 | 0.000 |  |  |  | (190) |  |
| Safi/Gath | 31.702 | 34.847 | Southern Levant | 1300 | 1200 | 1250 | 1500-1200 | 175 | 503 | 436 | 422 | 465 | 0.000 | 0.000 | 3.828 | 80.000 |  |  |  | (191) |  |
| Tel Burna | 31.630 | 34.874 | Southern Levant | 1300 | 1200 | 1250 | 1500-1200 | 254 | 486 | 421 | 408 | 449 | 2.765 | 23.000 | 2.074 | 23.000 |  |  |  | (192) |  |
| Tell Miqne_Ekron_Khirbet el-Muqanna | 31.779 | 34.850 | Southern Levant | 1300 | 1200 | 1250 | 1500-1200 | 104 | 528 | 457 | 443 | 488 | 0.000 | 0.000 | 0.000 | 0.000 |  |  |  | (193) |  |
| Jaffa | 32.054 | 34.753 | Southern Levant | 1300 | 1100 | 1200 | 1200-900 | 32 | 543 | 489 | 478 | 501 | 0.681 | 49.000 | 0.152 | 28.000 |  |  |  | (194) |  |
| Lachish | 31.565 | 34.849 | Southern Levant | 1300 | 1100 | 1200 | 1200-900 | 267 | 467 | 421 | 412 | 431 | 0.957 |  | 87.081 |  |  |  |  | (167) |  |
| Tell Tweini | 35.372 | 35.936 | Northern Levant | 1200 | 1100 | 1150 | 1200-900 | 26 | 756 | 682 | 666 | 699 | 10.283 | 71.000 | 30.077 | 100.000 |  |  |  | (135) |  |
| Nimrud | 36.099 | 43.329 | Mesopotamia | 1365 | 934 | 1149.5 | 1200-900 | 212 | 347 | 313 | 306 | 321 | 0.000 | 0.000 | 0.000 | 0.000 |  |  |  | (195) |  |
| Tell Schech Hamad | 35.646 | 40.743 | Mesopotamia | 1365 | 934 | 1149.5 | 1200-900 | 242 | 233 | 210 | 205 | 215 | 0.012 | 7.000 | 0.000 | 0.000 | 0.00 | 0.00 | 332 | (107) | (196) |
| Malyan | 30.050 | 52.420 | Iran | 1300 | 900 | 1100 | 1200-900 | 1618 | 254 | 229 | 224 | 235 | 0.000 | 0.000 | 0.000 | 0.000 | 0.00 | 0.00 | minimally 28 | (197) | (197) |
| Beth Shean | 32.504 | 35.503 | Southern Levant | 1200 | 1000 | 1100 | 1200-900 | -120 | 508 | 459 | 448 | 470 | 0.000 | 0.000 | yes | 60.000 | 75.00 | 0.00 | 52 | (198) | (199, 200) |
| Deir'Alla | 32.196 | 35.621 | Southern Levant | 1200 | 1000 | 1100 | 1200-900 | -219 | 438 | 395 | 386 | 405 | 0.001 | 20.000 | 20.000 | 0.000 | 12.95 | 0.00 | 6022 ml | (186) | (201) |
| Beer-Sheba_Tell es-Seba' | 31.245 | 34.841 | Southern Levant | 1200 | 1000 | 1100 | 1200-900 | 302 | 306 | 276 | 269 | 282 |  |  |  |  | 10.00 | 0.00 | 40 |  | (202) |
| Afula | 32.611 | 35.290 | Southern Levant | 1200 | 1000 | 1100 | 1200-900 | 65 | 613 | 553 | 540 | 566 | 0.000 | 0.000 | yes |  |  |  |  | (203) |  |
| Kamid el-Loz | 33.624 | 35.821 | Southern Levant | 1200 | 1000 | 1100 | 1200-900 | 934 | 710 | 640 | 626 | 656 | 0.038 | 33.000 | 0.000 | 0.000 |  |  |  | (180, 181) |  |
| Qubur al-Walaydah | 31.413 | 34.534 | Southern Levant | 1200 | 1000 | 1100 | 1200-900 | 79 | 340 | 307 | 300 | 314 | 3.401 | 7.000 | 0.170 | 1.000 |  |  |  | (204) |  |
| Sidon | 33.560 | 35.371 | Southern Levant | 1200 | 1000 | 1100 | 1200-900 | 20 | 826 | 745 | 728 | 763 | 6.977 | 43.000 | 17.054 | 100.000 |  |  |  | (178) |  |
| Tall al-'Umayri | 31.869 | 35.888 | Southern Levant | 1200 | 1000 | 1100 | 1200-900 | 904 | 307 | 277 | 270 | 283 | 7.273 |  | 0.000 | 0.000 |  |  |  | (185) |  |
| Shiloh | 32.056 | 35.290 | Southern Levant | 1150 | 1000 | 1075 | 1200-900 | 712 | 625 | 564 | 551 | 578 | 3.578 | 18.000 | 0.139 | 59.000 | 71.40 | 0.00 | 35 | (171) | (172) |
| Tell Qasile | 32.102 | 34.790 | Southern Levant | 1150 | 1000 | 1075 | 1200-900 | 14 | 578 | 510 | 487 | 534 | 0.534 | 11.000 | 0.000 | 0.000 | 0.00 |  |  | (205) |  |
| Tell Keisan | 32.873 | 35.151 | Southern Levant | 1150 | 1000 | 1075 | 1200-900 | 33 | 713 | 643 | 628 | 659 | 0.000 | 0.000 | 0.000 | 0.000 |  |  |  | (206) |  |
| Tell Shiukh Fawqani | 36.767 | 38.050 | Mesopotamia | 1200 | 918 | 1059 | 1200-900 | 345 | 447 | 403 | 394 | 413 | 0.000 | 0.000 | 0.000 | 0.000 | 0.00 | 0.00 | 667 | (95) | (17) |
| Tell Qarqur | 35.742 | 36.331 | Northern Levant | 1200 | 918 | 1059 | 1200-900 | 193 | 481 | 433 | 423 | 444 | 0.000 | 0.000 | 0.000 | 0.000 |  |  |  | (147) |  |
| Kenan Tepe | 37.831 | 40.813 | Mesopotamia | 1200 | 900 | 1050 | 1200-900 | 564 | 490 | 442 | 431 | 452 | 0.000 | 0.000 | 0.000 | 0.000 |  |  |  | (164) |  |
| Tell Afis | 35.905 | 36.799 | Northern Levant | 1200 | 900 | 1050 | 1200-900 | 360 | 356 | 322 | 314 | 329 | 1.784 | 17.000 | 1.499 | 26.000 |  |  |  | (42) |  |
| Tel Yoqneam_Tell Qemun_Jokneam | 32.665 | 35.109 | Southern Levant | 1200 | 900 | 1050 | 1200-900 | 99 | 675 | 609 | 595 | 623 | 0.000 | 0.000 | yes | 100.000 |  |  |  | (207) |  |
| Tell Aphek | 32.105 | 34.931 | Southern Levant | 1200 | 900 | 1050 | 1200-900 | 36 | 606 | 547 | 534 | 560 | 1.266 | 50.000 | 0.058 | 25.000 | yes |  |  | (184) | (184) |
| Timnah. Tel Batash | 31.784 | 34.911 | Southern Levant | 1200 | 900 | 1050 | 1200-900 | 129 | 540 | 487 | 476 | 499 |  |  | yes |  |  |  |  | (173) |  |
| Safi/Gath | 31.702 | 34.847 | Southern Levant | 1200 | 900 | 1050 | 1200-900 | 175 | 503 | 454 | 444 | 465 | 51.370 | 100.000 | 13.699 | 100.000 |  |  |  | (191) |  |
| Tell Halaf | 36.826 | 40.040 | Mesopotamia | 1150 | 900 | 1025 | 1200-900 | 363 | 396 | 357 | 349 | 366 |  |  |  |  | 0.00 | 0.00 | 318 |  | (54) |
| Kinet Höyük | 36.854 | 36.157 | Northern Levant | 1150 | 900 | 1025 | 1200-900 | 20 | 695 | 627 | 612 | 642 |  |  |  |  | 0.80 | 1.70 | 1189 |  | (54) |
| Tell Hesban | 31.801 | 35.809 | Southern Levant | 1200 | 800 | 1000 | 1200-900 | 889 | 344 | 310 | 303 | 318 | 8.333 | 33.000 | 0.000 | 0.000 |  |  |  | (208) |  |
| Khirbet al-Mudayna (el-'Aliya) | 31.281 | 35.871 | Southern Levant | 1050 | 950 | 1000 | 1200-900 | 794 | 164 | 148 | 145 | 152 | 2.420 | 28.000 | 0.000 | 0.000 |  |  |  | (209) |  |
| Feinan | 30.626 | 35.445 | Southern Levant | 1200 | 800 | 1000 | 1200-900 | 26 | 28 | 25 | 24 | 26 |  |  |  |  | 0.70 | 0.00 | 2257 |  | (210) |
| Tel Michal | 32.161 | 34.797 | Southern Levant | 1000 | 900 | 950 | 1200-900 | 14 | 608 | 548 | 536 | 562 | 50.000 |  | 0.000 | 0.000 |  |  |  | (179) |  |
| Pella | 32.450 | 35.613 | Southern Levant | 1100 | 800 | 950 | 1200-900 | -37 | 482 | 435 | 425 | 446 | 0.000 | 0.000 | 0.000 | 0.000 |  |  |  | (29) |  |
| Megiddo | 32.585 | 35.185 | Southern Levant | 1060 | 800 | 930 | 1200-900 | 192 | 644 | 581 | 568 | 595 | 0.323 |  | 72.698 | 67.000 | 81.60 | 0.00 | 449 | (73) | (74) |
| Horbat Rosh Zayit | 32.879 | 35.336 | Southern Levant | 1000 | 850 | 925 | 1200-900 | 202 | 676 | 610 | 596 | 624 | 0.006 | 75.000 | 0.000 | 0.000 | 57.10 | 0.00 | 21 | (211) | (212) |
| Ain Dara | 36.852 | 36.459 | Northern Levant | 1100 | 700 | 900 | 1200-900 | 219 | 668 | 602 | 589 | 617 | 21.268 | 77.000 | 0.000 | 0.000 |  |  |  | (213) |  |
| Tel Farah (South) | 31.282 | 34.483 | Southern Levant | 1200 | 586 | 893 | 1200-900 | 104 | 272 | 245 | 240 | 251 | 2.817 | 12.000 | 0.201 | 2.000 |  |  |  | (101) |  |
| Tell Abu al-Kharaz | 32.399 | 35.595 | Southern Levant | 1200 | 550 | 875 | 900-600 | -133 | 479 | 419 | 400 | 439 | 0.000 | 0.000 | 0.000 | 0.000 |  |  |  | (81) |  |
| Tell el Ifshar | 32.372 | 34.908 | Southern Levant | 1200 | 550 | 875 | 1200-900 | 20 | 670 | 604 | 590 | 619 | 0.000 | 0.000 | 0.000 | 0.000 |  |  |  | (153) |  |
| Tell Halif | 31.383 | 34.867 | Southern Levant | 1200 | 550 | 875 | 1200-900 | 483 | 391 | 352 | 344 | 361 | 0.000 | 0.000 | 0.000 | 0.000 |  |  |  | (40) |  |
| Hirbet el-Msas (Tel Masos) | 31.213 | 34.967 | Southern Levant | 1200 | 535 | 867.5 | 1200-900 | 357 | 318 | 287 | 280 | 294 | 16.667 |  | 0.000 | 0.000 |  |  |  | (38) |  |
| Tell Nebi Mend (Kadesh) | 34.556 | 36.519 | Northern Levant | 1200 | 535 | 867.5 | 1200-900 | 511 | 512 | 462 | 451 | 473 | 67.463 | 100.000 | 0.000 | 0.000 |  |  |  | (144) |  |
| Sidon | 33.560 | 35.371 | Southern Levant | 1000 | 700 | 850 | 900-600 | 20 | 826 | 723 | 689 | 757 | 6.186 | 75.000 | 25.086 | 100.000 |  |  |  | (178) |  |
| Tel Burna | 31.630 | 34.874 | Southern Levant | 1100 | 586 | 843 | 900-600 | 254 | 486 | 426 | 406 | 446 | 5.981 | 33.000 | 34.450 | 43.000 |  |  |  | (214) |  |
| Beer-Sheba_Tell es-Seba' | 31.245 | 34.841 | Southern Levant | 975 | 700 | 838 | 900-600 | 302 | 306 | 268 | 255 | 280 | 0.000 | 0.000 | 0.000 | 0.000 | 0.00 | 0.00 | 119 | (215) | (215) |
| Zincirli | 37.103 | 36.678 | Northern Levant | 975 | 700 | 837.5 | 900-600 | 501 | 604 | 528 | 504 | 553 |  |  |  |  | 1.20 | 0.70 | 3199 |  | (54) |
| Jiyeh/Porphyreon | 33.666 | 35.427 | Southern Levant | 925 | 750 | 837.5 | 900-600 | 54 | 860 | 753 | 718 | 788 | 0.000 | 0.000 | 57.143 | 71.000 |  |  |  | (216) |  |
| Tell Tweini | 35.372 | 35.936 | Northern Levant | 960 | 700 | 830 | 900-600 | 26 | 756 | 662 | 631 | 693 | 5.315 | 65.000 | 21.909 | 85.000 |  |  |  | (135) |  |
| Susa. Ville Royale | 32.189 | 48.258 | Iran | 1100 | 539 | 819.5 | 900-600 | 91 | 292 | 255 | 244 | 267 | 0.000 | 0.000 | 0.000 | 0.000 |  |  |  | (217) |  |
| Tappeh Gijlar | 37.930 | 45.300 | Iran | 1100 | 539 | 819.5 | 900-600 | 1267 | 345 | 302 | 288 | 316 | 50.000 | 100.000 | 0.000 | 0.000 |  |  |  | (44) |  |
| Beth Shean | 32.504 | 35.503 | Southern Levant | 900 | 700 | 800 | 900-600 | -120 | 508 | 445 | 424 | 466 | 0.000 | 0.000 | 9.803 | 33.000 |  |  |  | (218) | (200) |
| Kuntillet Ajrud | 30.186 | 34.428 | Southern Levant | 900 | 700 | 800 | 900-600 | 398 | 61 | 54 | 51 | 56 | 0.000 |  | yes | 41.000 | 0.00 | 0.00 | 146 | (219) | (219) |
| Safi/Gath | 31.702 | 34.847 | Southern Levant | 900 | 700 | 800 | 900-600 | 175 | 503 | 441 | 420 | 461 | 3.448 | 100.000 | 96.552 | 100.000 |  |  |  | (191) |  |
| Tell Khamîs | 36.718 | 38.186 | Mesopotamia | 900 | 700 | 800 | 900-600 | 26 | 429 | 375 | 358 | 393 | yes |  | 0.000 | 0.000 |  |  |  | (165) |  |
| Lachish | 31.565 | 34.849 | Southern Levant | 1000 | 586 | 793 | 900-600 | 267 | 467 | 409 | 390 | 428 | 1.911 | 20.000 | 87.898 | 20.000 | 58.70 | 0.00 | 341 | (167) | (167) |
| Tyros Al-Bass | 33.270 | 35.196 | Southern Levant | 1000 | 586 | 793 | 900-600 | 6 | 754 | 660 | 630 | 691 | 86.364 | 70.000 | 0.000 | 0.000 |  |  |  | (220) |  |
| Qubur al-Walaydah | 31.413 | 34.534 | Southern Levant | 1000 | 586 | 793 | 900-600 | 79 | 340 | 298 | 284 | 312 | 5.660 | 7.000 | 0.000 | 0.000 |  |  |  | (204) |  |
| Konar Sandal | 28.459 | 57.782 | Iran | 1100 | 480 | 790 | 900-600 | 577 | 51 | 45 | 42 | 47 | 0.000 | 0.000 | 0.000 | 0.000 | 5.10 | 0.00 | 350 | (115) | (115) |
| Nimrud | 36.099 | 43.329 | Mesopotamia | 934 | 612 | 773 | 900-600 | 212 | 347 | 304 | 290 | 318 | 0.000 | 0.000 | 0.000 | 0.000 |  |  |  | (195) |  |
| Tell Schech Hamad | 35.646 | 40.743 | Mesopotamia | 934 | 612 | 773 | 900-600 | 242 | 233 | 204 | 194 | 213 | 3.163 | 53.000 | 0.000 | 0.000 | 0.00 | 0.00 | 10139 | (107) | (196) |
| Megiddo | 32.585 | 35.185 | Southern Levant | 950 | 586 | 768 | 900-600 | 162 | 644 | 564 | 538 | 590 | 0.000 | 0.000 | 79.500 |  | 68.30 | 0.00 | 246 | (177) | (74) |
| Rifa'at | 36.470 | 37.130 | Northern Levant | 1200 | 332 | 766 | 1200-900 | 491 | 430 | 388 | 379 | 397 | 0.000 | 0.000 | 0.000 | 0.000 |  |  |  | (32) |  |
| Kinet Höyük | 36.854 | 36.157 | Northern Levant | 900 | 612 | 756 | 900-600 | 20 | 695 | 608 | 580 | 637 | 0.000 | 0.000 | 0.000 | 0.000 | 1.10 | 1.64 | 4634 | (221) | (54) |
| Tell Halaf | 36.826 | 40.040 | Mesopotamia | 900 | 600 | 750 | 900-600 | 363 | 396 | 347 | 331 | 363 |  |  |  |  | 0.00 | 1.70 | 931 | (101, 222) | (54, 222) |
| Tell Masaikh | 34.974 | 40.556 | Mesopotamia | 900 | 600 | 750 | 900-600 | 193 | 157 | 137 | 131 | 144 | 7.143 | 33.000 | 0.000 | 0.000 |  |  |  | (106) |  |
| Tell Halaf | 36.826 | 40.040 | Mesopotamia | 1200 | 300 | 750 | 1200-900 | 363 | 396 | 357 | 349 | 366 | 0.239 |  | 0.000 | 0.000 |  |  |  | (101) |  |
| Tell Qiri | 32.644 | 35.119 | Southern Levant | 1200 | 300 | 750 | 1200-900 | 62 | 675 | 609 | 595 | 623 | 0.000 | 0.000 | yes |  |  |  |  | (223) |  |
| Timnah. Tel Batash | 31.784 | 34.911 | Southern Levant | 900 | 586 | 743 | 900-600 | 129 | 540 | 473 | 451 | 495 |  |  | yes |  |  |  |  | (173) |  |
| Tepe Sialk | 33.969 | 51.405 | Iran | 918 | 535 | 726.5 | 900-600 | 956 | 158 | 138 | 132 | 145 | 6.509 | 25.000 | 0.000 | 0.000 | 0.00 | 0.00 | 574 | (11) | (12) |
| Tell Afis | 35.905 | 36.799 | Northern Levant | 918 | 535 | 726.5 | 900-600 | 360 | 356 | 312 | 297 | 327 | 2.525 | 75.000 | 0.000 | 0.000 |  |  |  | (42) |  |
| Tell Qarqur | 35.742 | 36.331 | Northern Levant | 918 | 535 | 726.5 | 900-600 | 193 | 481 | 421 | 401 | 440 | 17.315 | 75.000 | 0.000 | 0.000 |  |  |  | (147) |  |
| Qal'eh Ismail Aqa | 37.880 | 44.780 | Iran | 850 | 600 | 725 | 900-600 | 2060 | 464 | 407 | 388 | 425 | 0.000 | 0.000 | 0.000 | 0.000 |  |  |  | (44) |  |
| Tušhan (Ziyaret Tepe) | 37.793 | 40.793 | Mesopotamia | 900 | 540 | 720 | 900-600 | 566 | 489 | 428 | 408 | 448 | 6.441 |  | 0.000 | 0.000 | 0.00 | 0.00 | 962 | (224) | (26) |
| Bastam | 38.886 | 44.950 | Iran | 850 | 550 | 700 | 900-600 | 1182 | 350 | 306 | 292 | 320 | 0.244 | 10.000 | 0.000 | 0.000 | 0.00 | 0.00 | 63 | (225) | (226) |
| Tell el-Mazar II | 32.222 | 35.606 | Southern Levant | 800 | 586 | 693 | 900-600 | -239 | 453 | 396 | 378 | 415 | 0.001 | 25.000 | 0.000 | 0.000 |  |  |  | (227) |  |
| Patnos | 39.236 | 42.869 | Mesopotamia | 685 | 645 | 665 | 900-600 | 1639 | 527 | 461 | 439 | 482 | 0.000 | 0.000 | 0.000 | 0.000 |  |  |  | (228) |  |
| Tel 'Ira_Khirbet el-Garra | 31.233 | 34.986 | Southern Levant | 975 | 332 | 653.5 | 900-600 | 506 | 322 | 282 | 269 | 295 | 25.000 |  | 25.000 |  | 6.50 | 0.00 | 46 | (229) | (229) |
| City of David_Jerusalem | 31.772 | 35.235 | Southern Levant | 975 | 332 | 653.5 | 900-600 | 703 | 620 | 543 | 518 | 568 | 15.217 |  | 54.348 |  | 36.60 | 0.00 | 101 | (69) | (69) |
| Deir'Alla | 32.196 | 35.621 | Southern Levant | 700 | 586 | 643 | 900-600 | -219 | 438 | 384 | 366 | 402 | 0.214 | 54.000 | 0.028 | 20.000 | 7.50 | 0.00 | 2146 ml | (201) | (201) |
| Tell Burak | 33.482 | 35.322 | Southern Levant | 700 | 586 | 643 | 900-600 | 8 | 789 | 691 | 658 | 723 | 39.032 | 87.000 | 8.037 | 80.000 | 42.10 | 2.10 | 2629 | (230) | (54, 230) |
| Ashkelon | 31.664 | 34.547 | Southern Levant | 700 | 586 | 643 | 900-600 | 18 | 428 | 375 | 357 | 392 | 8.953 | 93.000 | 0.339 | 93.000 |  |  |  | (231) |  |
| Tell Shiukh Fawqani | 36.767 | 38.050 | Mesopotamia | 918 | 333 | 625.5 | 900-600 | 345 | 447 | 391 | 373 | 410 | 0.000 | 0.000 | 0.000 | 0.000 | 0.20 | 0.40 | 3351 | (31) | (17) |
| Nush-i Jan | 34.370 | 48.650 | Iran | 750 | 500 | 625 | 900-600 | 1685 | 478 | 418 | 399 | 438 | 0.143 | 10.000 | 0.000 | 0.000 |  |  |  | (232) |  |

*No values of this publication included in analysis

1. van Zeist W, Waterbolk-van Rooijen W. Chapter 10. The cultivated and wild plants. Tell Sabi Abyad The late Neolithic settlement. Leiden: National Museum of Antiquities; 1995. p. 521-50.

2. Caracuta V, Weiss E, van den Brink ECM, Liran R, Vardi J, Barzilai O. From natural environment to human landscape: New archaeobotanical data from the Neolithic Site of Nahal Zippori 3, Lower Galilee. Neo-Lithics 2014;1(14):33-41.

3. Allen SE. Palaeoethnobotany: Preliminary results. In: Garfinkel Y, Miller MAA, editors. Sha'ar Hagolan Vol 1, Neolithic art in context. Oxford: Oxbow Books; 2002. p. 235-46.

4. Hopf M. Jericho plant remains. In: Kenyon KM HT, editor. Excavations at Jericho V The pottery phases of the tell and other finds. London Council for British Archaeology; 1983. p. 576-621.

5. Kislev ME, Hartmann A. Food crops from Nahal Zehora II. In: Gopher A, editor. Village cmmunities of the Prepottery Neolithic period in the Menashe Hills, Israel Archaeological investigations at the sites of Nahal Zehora. Tel Aviv: Institute of Archaeology, Tel Aviv University; 2012. p. 1321-6.

6. Liphschitz N. Botanical analysis of wood remains from Nahal Zehora II. In: Gopher A, editor. Village communities of the Pottery Neolithic Period in the Menashe Hills, Israel Vol III. Monograph Series 2012. p. 1327-32.

7. Bernbeck R, Pollock S. The biography of an Early Halaf village: Fistikli Höyük 1999-2000. Istanbuler Mitteilungen 2003;53:9-77.

8. McCorriston J. The Halaf environment and human activities in the Khabur drainage, Syria. Journal of Field Archaeology 1992;19:315-33.

9. Neef R. Plant remains from archaeological sites in lowland Iraq: Tell el'Oueili In: Huot J-L, editor. 'Oueili Travaux de 1985. Paris: Editions Recherche sur les civilisations; 1991. p. 321-9.

10. van Zeist W, Bakker-Heeres JAH. Archaeobotanical studies in the Levant 2. Neolithic and Halaf levels at Ras Shamra. Palaeohistoria. 1984;26:151-70.

11. Tengberg M. Archaebotanical analysis at Tepe Sialk. Results from the 2003/2004 season. In: Shahmirzadi SM, editor. The Potters of Sialk. Sialk Reconsideration Project report 3. Tehran: Iranian Center for Archaeological Research; 2004.

12. Shirazi Z, Tengberg M. Vegetation and wood exploitation at Tape Sialk from the Neolithic to the Iron Age. In: Shahmirzadi SM, editor. The villagers of Sialk. Tehran: Iranian Center for Archaeological Research; 2012. p. 17-26.

13. Helbaek H. Samarran irrigation agriculture at Choga Mami in Iraq. Iraq. 1972;34:35-48.

14. Ekstrom H. Archaeobotanical remains from the 1998 and 1999 seasons at Tell Kurdu. Anatolica. 2000;26:80-3, 94.

15. Ekstrom H. Archaeobotanical report for the 2001 Tell Kurdu season. Anatolica 2004;30:69-70.

16. Willcox G. Chalcolithic carbonised cereals from Ubaid burnt storage structures at Kosak Shamali. In: Nishiaki Y, Matsutani T, editors. Tell Kosak Shamali Vol II. Tokyo: University Museum the University of Tokyo; 2003. p. 267-70.

17. Pessin H. Stratégies d'approvisionnement et utilisation du bois dans le Moyen Euphrate et la Damascène. Approche anthracologique comparative de sites historiques et préhistoriques. Unpublished PhD Thesis: Sorbonne University, Paris; 2004.

18. Miller NF. Palaeoethnobotanical results from Bendebal and Jaffarabad. Cahiers de la Délégation Archéologique Française en Iran 1983;13:277-84.

19. Jarl J, Riehl S, Deckers K, Becker JA. Plant cultivation under climatic fluctuations during the sixth and fifth millennia BC at Tell Tawila (northern Syria). Archaeological and Anthropological Sciences 2020;12:266.

20. Tosi M. Hasanlu project 1974: Palaeobotanical survey. Iran. 1975;13:185-6.

21. Graham P. Archaeobotanical remains from late 6th/early 5th millennium BC Tel Tsaf, Israel. Journal of Archaeological Science. 2014;43:105-10.

22. Liphschitz N. Timber in ancient Israel: dendroarchaeology and dendrochronology. Tel Aviv: Emery and Claire Yass Publications in Archaeology; 2007.

23. Langgut D, Garfinkel Y. 7000-year-old evidence of fruit tree cultivation in the Jordan Valley, Israel. Scientific Reports 2022;12:7463.

24. Holden T. The plant remains from Tell esh-Shuna North, Jordan Valley, unpublished ESH96 report. 1999.

25. Neef R. Introduction, development and environmental implications of olive culture: the evidence from Jordan. In: Bottema S, Entjes-Nieborg G, van Zeist W, editors. Man´s role in the shaping of the Mediterranean landscape. Rotterdam: Balkema; 1990. p. 295-306.

26. Proctor L., Smith A. Fuel and Plant Use in Northern Mesopotamia [Internet]. 2023 [cited 2023 Sept 27]. Available from: <https://doi.org/10.6078/M72Z13NS>.

27. Miller NF. The plant remains. In: Wright HT, editor. An early town on the Deh Luran plain, excavations at Tepe Farukhabad. Ann Arbor: University of Michigan Museum of Anthropology; 1981. p. 227-32, 427-30.

28. Meadows J. Early Farmers and their environment: archaeobotanical research at Neolithic and Chalcolithic sites in Jordan. Unpublished PhD: La Trobe University; 2005.

29. Willcox G. Archaeobotanical investigations at Pella (1983). In: McNicoll AW, Edwards PC, Hanbury-Tenison J, Hennssy JB, Potts TF, Smith RH, et al., editors. Pella in Jordan 2 The second interim report of the joint University of Sydney and College of Wooster excavations at Pella 1982-1985. Mediterranean Archaeology Supplement 2. Sydney: Meditarch; 1992. p. 253-6.

30. Neef R. Les activités agricoles et horticoles. In: Dollfus G, Kafafi Z, editors. Abu Hamid village du IVe millénaire de la vallée du Jourdain. Amman: Centre Culturel Français et. Département des Antiquités de Jordanie; 1988. p. 29-30.

31. Willcox G. Unpublished dataset [Internet]. [cited 2023 Sept 27]. Available from: http://perso.wanadoo.fr/g.willcox/

32. Hillman GC. The barleys from Iron Age Rifa'at. In: Matthers J, editor. The river Qoueiq, Northern Syria, and its catchment: studies arising from the Tell Rifa'at survey 1977-79. BAR International Series Oxford: British Archaeological Reports; 1981. p. 508 - 10.

33. Liphschitz N. Archaeobotanical investigations. In: Gopher A, Tsuk T, editors. The Nahal Qanah cave Earliest gold in the southern Levant. Tel Aviv: Tel Aviv University; 1996. p. 202-4.

34. Liphschitz N. Chapter 12. The archaeobotanical remains. In: van den Brink ECM, Gophna R, editors. Shoham (North) Late Chalcolithic Burial Caves in the Lod Valley Israel. 27. Jerusalem: Israel Antiquities Authority Publications Department; 2005. p. 151-3.

35. de Vartavan C. Preliminary report on the plant remains from Sataf. Levant. 1991;23:50-2.

36. Negbi M. The botanical finds at Tell Abu Matar. Israel Exploration Journal 1955;5:257-8.

37. Costantini L, Costantini-Biasini L. Agriculture in Baluchistan between the 7th and the 3rd millennium BC. Newsletter of Baluchistan Studies. 1985;2:16-30.

38. Liphschitz N, Waisel Y. Analysis of the botanical material. In: Fritz V, Kempinski A, editors. Ergebnisse der Ausgrabungen auf der Hirbet el-Msas (Tel Masos) 1972-1975. Wiesbaden: Harrassowitz; 1983. p. 208-13.

39. Kislev ME. Chalcolithic plant husbandry and ancient vegetation at Shiqmim. In: Levy TE, editor. Shiqmim I Studies concerning Chalcolithic societies in the northern desert, Israel. BAR international series 356. Oxford: British Archaeological Reports; 1987. p. 251-79, 548-63.

40. Laustrup M, Seger JD. Botanical remains from Tell Halif. Bulletin of the American Schools of Oriental Research 1990:23-4.

41. Helbaek H. Ancient crops in the Shahrzoor valley in Iraqi Kurdistan. Sumer. 1960;16:79-81.

42. Wachter-Sarkady C. Archaeobotanical investigations. In: Cecchini SM, Mazzoni S, editors. Tell Afis (Siria) Scavi sull'acropoli 1988 - 1992. Pisa: Edizioni ETS; 1998. p. 451-80.

43. Kepenski C. New evidence from Grai Resh, northern Iraq- the 2001 and 2002 seasons. A Pre-Uruk expansion site from the Late Chalcolithic period. Zeitschrift für Orient-Archäologie. 2011;4:47-85.

44. Costantini L, Biasini LC. I resti vegetali dei saggi a Qal'eh Ismail Aga e a Tappel Gijar. In: Pecorella PE, Salvini M, editors. Tra lo Zagros e l'Urmia, richerche storiche ed arqueologiche nell'Azerbaigian iraniano. Rome: Edizioni dell'Ateneo; 1984. p. 397-402.

45. van Zeist W, Bakker-Heeres JAH. Prehistoric and early historic plant husbandry in the Altinova Plain, southeastern Turkey. In: van Loon MN, editor. Korucutepe 1. Amsterdam: North Holland Publishing Company; 1975. p. 221-57.

46. Colledge S. Plants and people. In: Matthews R, editor. Excavations at Tell Brak Exploring an Upper Mesopotamian regional centre, 1994-1996. Cambridge: Oxbow; 2003. p. 389-416.

47. Hald MM. A thousand years of farming. Late Chalcolithic agricultural practices at Tell Brak in northern Mesopotamia. Oxford: British Archaeological Reports; 2008.

48. Charles M, Pessin H, Hald MM. Tolerating change at Late Chalcolithic Tell Brak: responses of an early urban society to an uncertain climate. Environmental Archaeology 2010;15(2):183-98.

49. van Zeist W, Waterbolk-van Rooijen W, Bottema S. Some notes on the plant husbandry of Tell Hammam et-Turkmen. Hammam et-Turkmen. In: van Loon MN, editor. Report of the University of Amsterdam's 1981-84 excavations in Syria II. Istanbul: Nederlands Historisch-Archeologisch Instituut te Istanbul; 1988. p. 705-15.

50. Pasternak R. Zwischenbericht über die Arbeiten an den botanischen Funden aus Oylum Höyük: Chalkolithische Fundschichten. Istanbuler Mitteilungen 1997;47:68-70.

51. Nesbitt M, Bates J, Hillman GC, Mitchell S. The Archaeobotany of Asvan: Environment & Cultivation in Eastern Anatolia from the Chalcolithic to the Medieval Period. Ankara: British Institute of Archaeology; 2017.

52. Willcox GH. A history of deforestation as indicated by charcoal analysis of four sites in eastern Anatolia. Anatolian Studies 1974;24:117-33.

53. Delle Donne M. Agricoltura, alimentazione e paleoambiente della Jazira siriana tra IV e III mill. a.C. Le evidenze da Tell Mozan. Università degli Studi di Napoli “L’Orientale”. Napoli: Unior Press; 2019.

54. Deckers K. Unpublished data.

55. Miller NF. Some plant remains from the 1996 excavation at Yarim Höyük. In: Yarim Höyük and the Uruk expansion. Anatolica 1998;24:79-80, 8.

56. Wright HT, Miller NF, Redding R. Time and process in an Uruk rural center. In: Barrelet M-T, editor. L'Archéologie de l’Iraq: du début de l'époque néolithique à 333 avant notre ère: perspectives et limites de l'interprétation anthropologique des documents. Paris: Colloques internationaux du Centre National de la Recherche scientifique; 1981. p. 265-84.

57. Charles M, Bogaard A. Third-millennium BC charred plant remains from Tell Brak. In: Oates D, Oates J, McDonald H, editors. Excavations at Tell Brak Nagar in the third millennium BC. London: British School of Archaeology in Iraq; 2001. p. 301-26.

58. Gophna R, Liphschitz N. The Ashkelon trough settlements in the Early Bronze Age I: new evidence of maritime trade. Tel Aviv 1996;23(2):143-53.

59. Melamed Y. Dry and charred grains from 'Afula - a taphonomic approach. Atiqot 1996;30:69-70.

60. Meadows J. Arid-zone farming in the fourth millennium BC: The plant remains from Wadi Fidan 4. In: Walmsey A, editor. Australians Uncovering Ancient Jordan: Fifty Years of Middle Eastern archaeology. Sydney: University of Sydney; 2001. p. 153-64.

61. Murray MA. Preliminary archaeobotanical report. In: Jerablus-Tahtani, 1992-4: Preliminary report. Levant. 1995;27:24-5.

62. Murray MA. Archaeobotanical remains, 1995. In: Jerablus-Tahtani, Syria, 1995: Preliminary report. Levant. 1996;28:20-1.

63. Wilkinson TJ, Deckers K. 2. The regional setting of Jerablus Tahtani. In: Peltenburg Eea, editor. Tell Jerablus Tahtani, Syria, I Mortuary practices at an Early Bronze Age fort on the Euphrates River. Levant Supplementary Series. Oxford: Oxbow; 2015. p. 13-23.

64. Miller NF. Appendix 1. Some archaeobotanical remains from the 1992 excavation season at Hacinebi Tepe. In: Mesopotamian-Anatolian interaction at Hacinebi, Turkey: Preliminary report on the 1992 excavations. Anatolica 1994;20:145-89.

65. Miller NF. Hacinebi Tepe 1993: Archaeobotanical report. American Journal of Archaeology 1996;100:248 - 57.

66. Willcox G. Plant remains. In: Helms SW, editor. Jawa, lost city in the Black Desert. London: Methuen; 1981. p. 247-8.

67. Müller-Neuhof B, Betts A, Wilcox G. Jawa, Northeastern Jordan: The first 14C dates for the early occupation phase. Zeitschrift für Orient-Archäologie 2015;8:124-31.

68. Western C. Appendix F. Catalogue of identified charcoal samples. In: Kenyon K, Holland TA, editors. Excavations at Jericho The pottery phases of the tell and other finds. London: The British Academy; 1983. p. 771-3.

69. Liphschitz N, Waisel Y. Macrobotanical remains. In: de Groot A, Ariel D, editors. Excavations at the City of David 1978-1985 Volume III Stratigraphical, environmental, and other reports. Jerusalem: University of Jerusalem; 1992. p. 105-21.

70. Riehl S, Deckers K. IX Plant remains. In: Badreshany K, Genz H, Sader H, editors. An Early Bronze Age site on the Lebanese coast - Tell Fadous-Kfarabida 2004 and 2005: Final report. Bulletin d'archéologie et d'architecture Libanaises 9. Beyrouth: Direction Générale des Antiquités; 2005/2007. p. 84-8.

71. Riehl S, Deckers K. The botanical finds from the 2007 and 2008 seasons of excavations. In: Genz H, Çakırlar C, Damick A, Jastrzębska E, Riehl S, Deckers K, et al., editors. Excavations at Tell Fadous – Kfarabida: Preliminary report on the 2009 seasons of excavations. BAAL. 13. Beyrouth: Direction Générale des Antiquités; 2009/2010. p. 110-6.

72. Genz H, Riehl S, Çakırlar C, Slim F, Damick A. Economic and political organization of Early Bronze Age coastal communities: Tell Fadous-Kfarabida as a case study. Berytus 2016;55:79-119.

73. Borojevic K. The archaeobotanical finds. In: Finkelstein I, Ussishkin D, Halpern B, editors. Megiddo IV The 1998-2002 Seasons Tel Aviv University, Sonia and Marco Nadler Institute of Archaeology 24. Tel Aviv: Emery and Claire Yass Publications in Archaeology; 2006. p. 519-41.

74. Benzaquen M, I. F, Langgut D. Vegetation history and human impact on the environs of Tel Megiddo in the Bronze and Iron Ages: A dendroarchaeological analysis. Tel Aviv 2019;46(1):42-64.

75. Liphschitz N. Plant economy and diet in the Early Bronze Age in Israel: A summary of present research. In: de Miroschedji P, editor. L' urbanisation de la Palestine à l'age du Bronze ancien Bilan et perspectives des recherches actuelles Actes du colloque d'Emmaüs, (20 - 24 octobre 1986). BAR International Series. 527. Oxford: British Archaeological Reports; 1989. p. 269-77.

76. Liphschitz N. The ecology of ´en Besor oasis: the archaeobotanical remains from the 4th millennium BC. In: Gophna R, editor. Excavations at 'En Besor. Tel Aviv: Ramot Publishing House, Tel Aviv University; 1995. p. 229-33.

77. Liphschitz N. Analysis of botanical remains from Tel Dalit. In: Gophna R, editor. Excavations at Tel Dalit An Early Bronze Age walled town in Central Israel. Tel Aviv: Ramot Publishing; 1996. p. 125-6.

78. Mabry JB, Donaldson ML. Early town development and water management in the Jordan Valley: Investigations at Tell el-Handaquq North. Aasor 1995;53:115-47.

79. Gregor H-J. Paläobotanische Untersuchungen zur antiken Pflanzenwelt des Hassek Höyük im Tal des oberen Euphrat und ein Versuch zur Rekonstruktion des dortigen Ökosystems für den Übergang von der späten Urukzeit zur frühen Bronzezeit. In: Behm-Blancke MR, editor. Hassek Höyük Naturwissenschaftliche Untersuchungen und lithische Industrie. Istanbuler Forschungen Tübingen: Ernst Wasmuth Verlag; 1992. p. 34-57.

80. White CE, Chesson MS, Schaub RT. A recipe for disaster: Emerging urbanism and unsustainable plant economies at Early Bronze Age Ras an-Numayra, Jordan. Antiquity. 2014;88:363-77.

81. Holden TG. The charred plant remains from Tell Abu Kharaz in the Jordan Valley (AOC 1010). Edinburgh: AOC; 1994.

82. Chernoff MC, Paley SM. Dynamics of cereal production at Tell el Ifshar, Israel during the Middle Bronze Age. Journal of Field Archaeology 1988;25(4):397-416.

83. Costantini L, Dyson RH. The ancient agriculture of the Damghan plain: the archaeobotanical evidence from Tepe Hissar. In: Miller NF, White C, editors. Economy and settlement in the Near East Analyses of ancient sites and materials. Philadelphia: PA, MASCA, University Museum; 1990. p. 46-68.

84. Hopf M. Plant remains, Strata V-I. Early Arad: The Chalcolithic settlement and Early Bronze city. In: Amiran R, editor. Early Arad I The Chalcolithic settlement and Early Bronze city. Jerusalem: Israel Exploration Society; 1978. p. 64-82.

85. Hopf M. Plant remains, Strata V-I. In: Amiran R, editor. Early Arad: The Chalcolithic settlement and Early Bronze city. Jerusalem: Israel Exploration Society; 1978. p. 64–82.

86. Liphschitz N. Notes on the archaeobotanical remains. In: Greenberg R, editor. Tel Beth Yerah. The Early Bronze Age mound. Volume II. Urban Structure and Material Culture 1933-1986 Excavations IAA Reports Jerusalem: Israel Antiquities Authority Publications Department; 2014. p. 303-4.

87. McCreery DW. The paleoethnobotany of Bab edh-Dhra. In: Rast WE, Schaub R, editors. Bab adh-Dhra Excavations at the Town Site (1975-1981. Winona Lake, Indiana: Eisenbrauns; 2003. p. 449-63.

88. Bourke S, Sparks R, McLaren B, Sowada K, Mairs L, Meadows J, et al. Preliminary report on the University of Sydney's eighteenth and nineteenth seasons of excavations at Pella (Tabaqat Fahl) in 1996/1997. Annual of the Department of Antiquities of Jordan 2003;47:335-3488.

89. Simchoni O, Kislev ME. Chapter 12A: Food and fodder in Early Bronze Age strata in Area M. In: Mazar A, editor. Excavations at Tel Beth Shean 1989-1996 Volume IV The 4th and 3rd Millennia BCE. Jerusalem: The Israel Exploration Society; 2012. p. 422-9.

90. Baruch U. Chapter 12B: Wood remains from area M. In: Mazar A, editor. Excavations at Tel Beth-Shean 1989-1996. Jerusalem: The Israel Exploration Society; 2012. p. 430-1.

91. Costantini L, Biasini LC. The plant remains. In: Wilhelm G, Zaccagnini C, editors. Tell Karrana 3 Tell Jikan Tell Khirbet Salih. Baghdader Forschungen1993. p. 237-50.

92. Miller NF. Palaeoethnobotanical evidence for deforestation in ancient Iran: A case study of urban Malyan. Journal of Ethnobiology 1985;5(1):1-19.

93. Salavert A. Olive cultivation and oil production in Palestine during the Early Bronze Age (3500 - 2000 B.C.): the case of Tel Yarmouth, Israel. Vegetation History and Archaeobotany 2008;17(1):S53-S61.

94. Strange J. Tall al-Fukhār. Results from the excavations in 1990-93 and 2002. Volume I. Text Aarhus: Aarhus University Press; 2015.

95. Klesly G. Unpublished dataset. Charred plant remains from sites on the Euphrates.

96. Hillman GC. Agricultural resources and settlement in the Asvan Region. Anatolian Studies 1973;23:217-24.

97. Ilgezdi G. The domestication process in southeastern Turkey: The evidence of Mezraa-Teleilat. Unpublished M.Sc. thesis: University of Tübingen; 2008.

98. Helbaek H. Plant economy in ancient Lachish. In: Tufnell O, editor. Lachish IV: The Bronze Age. Oxford: Oxford University Press; 1958. p. 309-17.

99. Follieri M, Coccolini GBL. Palaeoethnobotanical study of the VI A and VI B periods of Arslantepe (Malatya, Turkey). Preliminary report. Origini 1983;12(2):599-617.

100. Masi A, Restelli FB, Sabato D, Vignola C, Sadori L. Timber exploitation during the 5th–3rd millennia BCE at Arslantepe (Malatya, Turkey): Environmental constraints and cultural choices. Archaeological and Anthropological Sciences. 2018;10:465–83.

101. Riehl S. unpublished data.

102. Oybak Dönmez E. Prehistoric and Medieval plant remains from two sites on the Euphrates, south-eastern Turkey. Turkish Journal of Botany 2006;30:11-38.

103. Samuel D. Archaeobotanical work at Tell Zagan and Tell Hwes, 1987. In: The AUB-IFEAD Habur village project preliminary report: rural settlement in the Syrian Gazira from prehistoric to modern times. Damaszener Mitteilungen 1989;4:19-23.

104. Miller NF. Sweyhat and Hajji Ibrahim: Some archaeobotanical samples from the 1991 and 1993 seasons. In: Zettler RL, editor. Subsistence and settlement in a marginal environment: Tell es-Sweyhat, 1989 - 1995. Philadelphia: University of Pennsylvania Museum of Archaeology and Anthropology; 1997. p. 95–112.

105. McCreery DW. Flotation of the Bab edh-Dhra and Numeira plant remains. Annual of the American School of Oriental Research 1981;46:165-9.

106. Kubiak-Martens L. Plant remains from Tell Ashara (Terqa) and Tell Masaikh in the Middle Euphrates, south-eastern Syria. Archaeobotanical report (field seasons 2006 and 2007). PURATTIM. 2015;3:423-42.

107. van Zeist W. Third to first millennium BC plant cultivation on the Khabur, North-Eastern Syria. Palaeohistoria. 1999/2000 (2001);41/42:111-25.

108. De Moulins D. Sidon grain from the store-rooms. Archaeology and History in the Lebanon. 2009;29:11-5.

109. Allué E, Griffiths D. Identification of wood from Bronze Age contexts at Sidon. Archaeology and History in the Lebanon 2006;24:23-33.

110. Cichocki O. Synopsis of investigations on Sidon charcoal finds. Archaeology and History in the Lebanon. 2019;48-49:123-9.

111. Field H. Ancient wheat and barley from Kish, Mesopotamia. American Anthropologist 1932;4(2):303-9.

112. Charles M. Botanical remains. In: Green A, editor. Abu Salabikh excavations Volume 4 The 6G ash-tip and its contents: cultic and administrative discard from the temple? . London: British School of Archaeology in Iraq; 1993. p. 203-7.

113. McCorriston J. Preliminary archaeobotanical analysis in the Middle Habur valley, Syria and studies of socioeconomic change in the early third millennium BC. Bulletin of the Canadian Society for Mesopotamian Studies. 1995;29:33-46.

114. McCorriston J. Cultural and environmental history in archaeological charred woods from the Khabur drainage, Upper Mesopotamia. Varia Anatolica 2007;19:503-22.

115. Mashkour M, Tenberg M, Shirazi Z, Madjidzadeh Y. Bio-archaeological studies at Konar Sandal, Halil Rud basin, southeastern Iran. Journal of Environmental Archaeology. 2013;18(3):222-46.

116. van Zeist W, Bakker-Heeres JAH. Archaeobotanical studies in the Levant 4. Bronze Age sites on the north Syrian Euphrates. Palaeohistoria. 1985;27:247-316.

117. Moffett L. Appendix. Early Bronze Age plant remains from Tell Nebi Mend. A preliminary report. Levant 1989;21:29-32.

118. van Zeist W. Some notes on second millennium BC plant cultivation in the Syrian Jazira. Leuven: Peeters; 1994.

119. Engel T. Archaeobotanical analysis of timber and firewood used in the third millennium houses at Tall Bderi/Northeast Syria. In: Veenhof KR, editor. Houses and Households. Leiden: Nederlands Instituut voor het Nabije Oosten; 1993. p. 105-13.

120. Kabukcu C. Early agriculture in northern Syria: botanical remains from Jerablus Tahtani. Unpublished Master thesis: Trent University; 2012.

121. Miller NF. Vegetation and land use. Vegetation and land use. In: The Chicago Euphrates Archaeological Project. Anatolica 1986;13:85-9, 119-20.

122. Miller NF. Sweeter than wine? The use of grape in early western Asia. Antiquity. 2008;82:937-46.

123. Algaze G, Ataman K, Ingraham M, Marfoe L, McDonald M, Miller N, et al. The Chicago Euphrates Archaeological Project 1980-1984: An interim report. Anatolica. 1986;XIII:83-121.

124. Riehl S. Resource management at Tell el-'Abd. Economic specialization despite or because of environmental limitations? . In: Finkbeiner U, editor. Final reports of the Syrian/German excavations at Tell el/Abd, Volume III. Marru. Münster: Zaphon; 2019. p. 169-85.

125. Deckers K. The charcoal remains from third millennium BC Tell el-'Abd. In: Finkbeiner U, editor. Final Reports of the Syrian/German Excavations at Tell el/Abd. Marru. III. Münster: Zaphon; 2019. p. 187-92.

126. Wachter-Sarkady C. Consuming plants. Archaeobotanical samples from Royal Palace G and Building P4. In: Matthiae P, Marchetti N, editors. Ebla and its Landscape Early State Formation in the Ancient Near East. New York: Routledge; 2013. p. 382-408.

127. Fiorentino G, Caracuta V. Use of wood and environment in Bronze Age Ebla (NW Syria): results of the anthracological analysis. In: Damblon F, editor. Proceedings of the Fourth International Meeting of Anthracology Brussels, 8-13 September 2008, Royal Belgian Institute of Natural Sciences. Bar International Series Oxford: British Archaeological Reports; 2013. p. 93-102.

128. Caracuta V, Fiorentino G. Chapter 24. Forests near and far. An anthracological perspective on Ebla. In: Matthiae P, Marchetti N, editors. Ebla and its landscape Early state formation in the Ancient Near East. Walnut Creek: Left Coast Press; 2013. p. 403-12.

129. Porson S, Fall PL, Falconer SE. Archaeobotanical analyses of carbonised plant remains from Tell Abu en-Ni‘aj. In: Falconer SE, Fall PL, editors. Early Bronze IV Village Life in the Jordan Valley Excavations at Tell Abu en-Ni'aj and Dhahret Umm el-Marar, Jordan. BAR International Series Oxford: BAR Publishing; 2019. p. 131-8.

130. Herveux L. Étude archéobotanique préliminaire de tell al-Rawda, site de la fin du Bronze ancien en Syrie intérieure. Akkadica. 2004;1:79-91.

131. Matilla Séiquer G, Rivera Núñez D. Estudio paleoetnobotánico de Tell Qara Quzaq-I. In: del Olmo Lete G, editor. Tell Qara Quzaq-I Campañas I-III (1989-1991). Barcelona: Editorial Ausa; 1994. p. 151-82.

132. Pena-Chocarro L, Rottoli M. Crop husbandry practices during the Bronze and Iron Ages in Tell Mishrifeh (Central-Western Syria). In: D. MB, editor. Urban and natural landscapes of an ancient Syrian capital Settlement and environment at Tell Mishrife/Qatna and in Central Western Syria Proceedings of the International Conference held in Udine 9-11 December 2004 (= Studi archeologici su Qatna 1. Documents d'archéologie syrienne. 12. Udine2007. p. 113-33.

133. Riehl S. Plant production at Qatna in the environmental and supra-regional economic context. In: Pfälzner P, editor. Qatna and the networks of Bronze Age globalism Akten einer internationalen Konferenz in Stuttgart im Oktober 2009. Qatna Studien Supplementa. Wiesbaden: Harrassowitz; 2015. p. 477-86.

134. Riehl S, Deckers K. Die Umwelt des Königspalastes: Eine Synthese der archäobiologischen Untersuchungen. In: Pfälzner P, Schmid J, editors. Der Königspalast von Qatna Teil 1: Chronologie, Grundriss, Baugeschichte und Bautechniken Qatna Studien 5. Wiesbaden: Harrassowitz; 2019. p. 79-87.

135. Linseele V, Marinova E, De Cupere B, Van der Valk J, Vandorpe P, Van Neer W. Bronze and Iron Age Palaeo-economy in a changing environment. The bioarchaeology of Tell Tweini on the northern Levantine coast. In: Bretschneider J, Jans G, editors. About Tell Tweini (Syria): artefacts, ecofacts and landscape Research results. Leuven: Peeters; 2019. p. 417-618.

136. Riehl S. Maintenance of agricultural stability in a changing environment - the archaeobotanical evidence at Emar. In: Finkbeiner U, Sakal F, editors. Emar after the closure of the Tabqa Dam The Syrian-German Excavations 1996-2002 Volume I: Late Roman and Medieval Cemeteries and Environmental studies. Subartu. 25. Brepols: Turnhout; 2010. p. 177-224.

137. Deckers K. Anthracological research at the archaeological site of Emar on the Middle Euphrates, Syria. Paléorient 2005;32(2):152-66.

138. Deckers K. Vegetation and wood use in the Bronze Age based on charcoals from Emar. In: Finkbeiner U, Sakal F, editors. Emar after the closure of the Tabqa Dam The Syrian-German Excavations 1996-2002 Volume I: Late Roman and Medieval Cemeteries and Environmental studies. 25. Turnhout: Brepols; 2010. p. 225-44.

139. Wetterstrom W. Ninevite 5 period agriculture at Tell Leilan: Preliminary results. In: Rova E, Weiss H, editors. The origins of north Mesopotamian civilization: Ninevite 5 chronology, economy, society Subartu. Turnhout: Brepols; 2003.

140. Deckers K, Pessin H. Vegetation development in relation to human occupation and climatic change in the Middle Euphrates and Upper Jazirah (Syria/Turkey) during the Bronze Age. Quaternary Research 2010;74:216-26.

141. Riehl S. Plant production in a changing environment: the archaeobotanical remains from Tell Mozan. In: Deckers K, Doll M, Pfälzner P, Riehl S, editors. Development of the environment, subsistence and settlement of the city of Urkeš and its region. Harrassowitz: Wiesbaden; 2010. p. 13-158.

142. Deckers K. Anthracological research at Tell Mozan. In: Deckers K, Doll M, Pfälzner P, Riehl S, editors. Development of the environment, subsistence and settlement of the city of Urkeš and its region. Wiesbaden: Harrassowitz; 2010. p. 361-78.

143. Waines JG. Plant remains from Tell Taya, Iraq. Iraq. 1973;35:185-7.

144. Walker A. Bronze Age and Iron Age charred plant remains from Tell Nebi Mend, Syria. MSc Thesis: University of Sheffield; 2008.

145. Ellison R, Renfrew J, Brothwell D, Seeley N. Some food offerings from Ur, excavated by Sir Leonard Woolley, and previously unpublished. Journal of Archaeological Science. 1978;5:167-77.

146. Falconer SE, Fall PL. Bronze Age rural ecology and village life at Tell el-Hayyat, Jordan. Oxford: Archaeopress; 2006.

147. Smith A. Climate, culture, and agriculture: examining change in the Near East during the Bronze and Iron Ages. Unpublished PhD Dissertation: Boston University; 2005.

148. Oybak E, Demirci S. Early Bronze Age plant remains from Imamoglu Höyük, SE Turkey. Anatolian Studies 1997;47:173-21.

149. Schwartz GM, Curvers HH, Gerritsen FA, MacCormack JA, Miller NF, Weber JA. Excavation and survey in the Jabbul Plain, Western Syria: The Umm el-Marra project 1996-1997. American Journal of Archaeology. 2000;104(3):419-62.

150. Wasylikowa K, Kolinski R. The role of plants in the economy of Tell Arbid, north-east Syria, in the Post-Akkadian period and Middle Bronze Age. Acta Palaeobotanica 2013;53(2):263–93.

151. Riehl S, Orendi A. Archaeobotanical samples from Middle and Late Bronze Age contexts at Tell el-Burak. In: Kamlah J, Sader H, editors. Tell el-Burak I The Middle Bronze Age. Abhandlungen des Deutschen Palästina-Verein. Wiesbaden: Harrassowitz; 2019. p. 360-8.

152. Deckers K. Charcoal analysis results from Middle Bronze Age monumental building contexts at Tell el-Burak. In: Kamlah J, Sader H, editors. Tell el-Burak I The Middle Bronze Age. Abhandlungen des Deutschen Palästina-Vereins Wiesbaden: Harrassowitz; 2019. p. 269-380.

153. Chernoff MC. The archaeobotanical material from Tel el Ifshar, Israel: a diachronic study of agricultural strategies during the third and second millennia B.C.E. Brandeis: Brandeis University; 1998.

154. Berelov B. Occupation and abandonment of Middle Bronze Age Zahrat adh-Dhra’ 1, Jordan. The behavioural implications of quantitative ceramic analyses. Oxford: British Archaeological Reports; 2006.

155. Deckers K. Anthracological research on charcoal samples from Atchana. In: Yener KAS, editor. Tell Atchana, Ancient Alalakh The 2003-2004 Excavations Seasons. Istanbul: Koç University Press; 2010. p. 137-9.

156. Miller NF. Plant remains. In: Excavations and survey in the Jabbul Plain, Western Syria: The Umm el-Marra Project 1996-1997. American Journal of Archaeology 2000;104(3):438-47.

157. Caracuta V, Fiorentino G. Garbage disposal in the Middle Bronze Age in Tell Mardikh-Ebla (NW Syria): Using plant remains to investigate midden formation processes. In: Baffi F, Fiorentino G, Peyronel L, editors. Tell Tuqan excavations and regional perspectives Cultural developments in inner Syria from the Early Bronze Age to the Persian/Hellenistic period Proceedings of the International Conference May 15th-17th 2013 Lecce. Galatina: Congedo Editore; 2014. p. 485-506.

158. Nesbitt M, Samuel D. Archaeobotany in Turkey: A review of current research. Orient Express 1996;3:91 - 6.

159. Hynd A. A model of local continuity: The 1995 archaeobotanical assemblage from Kinet Höyük, Hatay. Unpublished dissertation: University of Sheffield; 1997.

160. Genz H, van Ess M, Riehl S, Deckers K, Höflmayer F. Baalbek in the Bronze Ages: Preliminary results of a sounding in the courtyard of the Jupiter Temple in 2012 and 2019. BAAL. 2023;23:155-205.

161. De Moulins D. Sidon: Plant remains from the Middle Bronze Age. In: Doumet-Serhal C, Rabate A, Resek A, editors. And Canaan begat Sidon his firstborn, a tribute to Dr John Curtis on his 65th Birthday. Archaeology and History in the Lebanon. Beirut: The Lebanese British Friends of the National Museum, Beirut; 2011. p. 34-5.

162. van Zeist W. Palaeobotanical investigations of Tell ed-Der. In: de Meyer L, editor. Tell ed-Der IV Progress reports. Leuven: Peeters; 1984. p. 119-33.

163. Deckers K. Anthracological samples from the Early and Middle Bronze Age graves at Chagar Bazar (Syria). In: Tunca Ö, Baghdo AM, editors. Chagar Bazar (Syrie) VIII Les tombes ordinaires de l’âge du Bronze ancien et moyen des chantiers D-F-H-I (1999-2011) Études diverses. Louvain: Peeters; 2016. p. 49-53.

164. Ekstrom H. Archaeobotanical remains. Anatolica. 2003;29:121-5.

165. Valera J, Matilla-Seiquer G, Obón C, Rivera D. Archaeobotanical study of Tell Khamîs (Syria). Heritage. 2022;5:1687-718.

166. Kislev ME. The relative impact of pig husbandry versus goat browsing on ancient oak forests in Israel. In: Edelstein G, Milevski I, Aurant S, editors. The Rephaim Valley Project Villages, terraces, and stone mounds Excavations at Manahat, Jerusalem, 1987-1989. Jerusalem: Israel Antiquities Authority; 1998. p. 113 - 8.

167. Liphschitz N. The archaeobotanical remains. In: Ussishkin D, editor. The renewed archaeological excavations at Lachish 1973-1994. Tel Aviv: Emery and Claire Yass Publications in Archaeology; 2004. p. 2230-47.

168. Simchoni O, Kislev ME, Melamed Y. Chapter 15A: Beth-Shean as a trade center for crops in the Bronze Age: Botanical and entomological evidence. In: Mazar A MR, editor. Excavations at Tel Beth Shean 1989-1996 Volume II The Middle and Late Bronze Age strata in Area R. Jerusalem: The Israel Exploration Society; 2007. p. 702-15.

169. Baruch U. Chapter 15: Identification of wood remains from Area R. In: Mazar A, Mullins RA, editors. Excavations at Tell Beth-Shean 1989-1996 Vol II The Middle and Late Bronze Age strata in area R. Jerusalem: Israel Exploration Society; 2007. p. 716-7.

170. Deckers K, Karakaya D, Poolman L, Ögüt B, Herrmann J, Morgan KR. An estate at Zincirli? Land use and resource exploitation at the Middle Bronze Age monumental building Complex DD in Zincirli, Gaziantep Province of Turkey. Archaeological and Anthropological Sciences 2023;15:13.

171. Kislev ME. Chapter 17. Food remains. In: Finkelstein I, editor. The archaeology of a biblical site. Monograph Series of the Institute of Archaeology Tel Aviv University Tel Aviv: Institute of Archaeology of Tel Aviv University, Publications Section; 1993. p. 354 - 61.

172. Liphschitz N. Palaeobotanical remains. In: Finkelstein I, Bunimovits S, Lederman Z, editors. Shiloh, the archaeology of a biblical site. Tel Aviv: Institute of Archaeology of Tel Aviv University; 1993. p. 351-3.

173. Kislev ME, Melamed Y, Langsam Y. Plant Remains from Tel Batash. In: Panitz-Cohen N, Mazar A, editors. Timnah (Tel Batash) III: The finds from the second millenium BCE. Qedem Monographs of the Institute of Archaeology. 45. Jerusalem: The Hebrew University of Jerusalem; 2006. p. 295-310.

174. Riehl S. Flourishing agriculture in times of political instability: The archaeobotanical and isotopic evidence from Tell Atchana. In: Yener KAS, editor. Tell Atchana, ancient Alalakh The 2003-2004 excavations seasons. Istanbul: Koç University Press; 2010. p. 123-36.

175. Küster H. Bronzezeitliche Pflanzenreste aus Tall Munbaqa. Mitteilungen der Deutschen Orient-Gesellschaft zu Berlin 1989;121:85-91.

176. Deckers K. Die Auswertung der Holzkohlenproben aus Raum DK. In: In Wissing A. GE, Lange-Weber S. , editor. Der Königspalast von Qatna Teil 3: Architektur, Stratigraphie, Funde und Keramik der westlichen, nördlichen und östlich-zentralen Raumeinheiten Die Nutzungsphasen G 8 bis G5/6. 11.3. Wiesbaden: Harrassowitz; in press. p. 101-23.

177. Liphschitz N. Wood remains. In: Finkelstein I, Ussishkin D, Cline EH, editors. Megiddo V: The 2004-2008 seasons. 31. Tel Aviv: Tel Aviv University; 2013. p. 1220-36.

178. De Moulins D. Plant Remains from Middle Bronze Age to Iron Age. Samples of the College Site, Sidon. Archaeology and History in Lebanon 2015;42-43:32-54.

179. Liphschitz N, Waisel Y. Botanical remains. In: Herzog Z, Rapp G, Negbi O, editors. Excavations at Tel Michal. Minneapolis: University of Minnesota Press; 1989. p. 219-22.

180. Behre K-E. Kulturpflanzenreste aus Kamid el-Loz. In: Hachmannn R, editor. Bericht über die Ergebnisse der Ausgrabungen in Kamid el-Loz (Libanon) in den Jahren 1966 und 1967. Bonn: Rudolf Habelt Verlag; 1970. p. 59-69.

181. Baas J. Ein bedeutsamer botanischer Fund der Gattung Echium Linne aus Kamid el-Loz. Bericht über die Ergebnisse in Kamid el-Loz in den Jahren 1968 bis 1970. Natur und Museum 1977;107:78-82.

182. Baruch U. Wood remains. In: Panitz-Cohen N, Mazar A, editors. Excavations at Tel Beth Shean (1989-1996): The 13th-11th century BCE strata in Areas N and S Vol III Jerusalem: Israel Exploration Society; 2009. p. 772-3.

183. Kislev ME, Mahler-Slasky Y. Food Remains. In: Gadot Y, Yadin E, editors. Aphek-Antipatris III (Tel Aviv). Tel Aviv: Institute of Archaeology, Tel Aviv University; 2009. p. 499-525.

184. Liphschitz N. Archaeobotanical remains. In: Gadot Y, Yadin E, editors. Aphek-Antipatris III (Tel Aviv). Monograph Series 27. Tel Aviv: Tel Aviv University; 2009. p. 493-8.

185. Ramsay J, Mueller N. Telling seeds: archaeobotanical investigations at Tall al-‘Umayri, Jordan. In: McGeough KM, editor. The archaeology of agro-pastoralist in Jordan. The Annual of the American Schools of Oriental Research. Boston MA: American Schools of Oriental Research; 2016. p. 1-25.

186. van Zeist W, Bakker-Heeres JAH. Paleobotanical studies of Deir `Alla, Jordan. Paléorient. 1973;1:21-37.

187. Nicolì M, Riehl S, Webster L, Streit K. Agricultural resources in the Bronze Age city of Tel Lachish. Vegetation History and Archaeobotany 2022;31:559-77.

188. Melamed Y. Botanical remains from the excavation on the southern beach of Ashdod. Atiqot 2013;74:127−31.

189. Kislev ME. Cereal grains from a pit. In: Dothan E, Brandl B, editors. Deir El-Balah: Excavations in 1977-1982 in the cemetery and settlement Volume II, The Finds. 49. Jerusalem: The Hebrew University of Jerusalem; 2010. p. 307-8.

190. Riehl S. Archäobotanische Funde aus der Kampagne 2009 in Qubur al-Walaydah. Welt des Orients 2010;40:259–67.

191. Mahler-Slasky Y, Kislev ME. Preliminary archaeobotanical research at Tell es-Safi/Gath - The 1997-2002 Seasons. In: Maeir AM, editor. Tell es-Safi/Gath I - The 1996-2005 Seasons, Volume I. Ägypten und Altes Testament. 69. Wiesbaden: Harrassowitz; 2012. p. 579-87.

192. Riehl S, Shai I. Supra-regional trade networks and the economic potential of Iron Age II sites in the southern Levant. Journal of Archaeological Science: Reports 2015;3:525-33.

193. Mahler-Slasky Y, Kislev ME. Lathyrus consumption in Late Bronze and Iron age sites in Israel: An Aegean affinity. Journal of Archaeological Science 2010;37:2477-85.

194. Orendi A. unpublished data.

195. Helbaek H. The plant remains from Nimrud. In: Mallowan ME, editor. Nimrud and its remains. London: Collins; 1966. p. 613-20.

196. Frey W, Jagiela C, Kürschner H. Holzkohlefunde in Dur-Katlimmu/Tall Seh 588 Hamad und ihre Interpretation. In: Kühne H, editor. Die rezente Umwelt von Tall 589 Seh Hamad und Daten zur Umweltrekonstruktion der assyrischen Stadt Dūr-Katlimmu. Berlin: Dietrich Reimer Verlag; 1991. p. 137-61.

197. Miller NF. Palaeoethnobotany. In: Carter E, editor. Excavations at Anshan (Tal-e Malyan): the Middle Elamite period. Philadelphia: University Museum; 1996. p. 99-108.

198. Kislev ME, Simchoni O, Melamed Y, Maroz L. Food and industrial crops. In: Panitz-Cohen N MA, editor. Excavations at Tel Beth Shean 1989-1996 Volume III The 13th-11th century BCE strata in area N and S. Jerusalem: Israel Exploration Society; 2009. p. 764-71.

199. Baruch U. Chapter 25. Identification of wood remains from areas Q, S and P. In: Mazar A, editor. Excavations at Tel Beth-Shean 1989-1996 Vol I From the Late Bronze Age IIB to the Medieval period. Jerusalem: The Israel Exploration Society; 2006. p. 687-8.

200. Baruch U. Chapter 17B: Wood remains. In: Mazar A, editor. Excavations at Tel Beth-Shean 1989-1996 Vol I From the Late Bronze Age IIB to the Medieval period. Jerusalem: The Israel Exploration Society; 2006. p. 772-3.

201. van Zeist W, Heeres JAH. Paleobotanical studies of Deir´Alla, Jordan. Paléorient. 1973;1:21-37.

202. Liphschitz N. Chapter seven. The botanical material of Iron Age I. In: Herzog Z, editor. Beer-Sheba II The Early Iron Age settlements. Tel Aviv: Tel Aviv University; 1984. p. 116-7.

203. Zaitschek DV. Remains of cultivated plants from Afula. Atiqot. 1955;1:71-4.

204. Orendi A, Riehl S. Unpublished data.

205. Kislev ME, Hopf M. Food Remains from Tell Qasile, with Special Reference to *Lathyrus sativus/cicera*. In: Mazar A, editor. Excavations at Tell Qasile, part 2. 20. Jerusalem: The Hebrew University of Jerusalem; 1985. p. 140-8.

206. Kislev ME. Contenu d'un silo à blé de l'époque du fer ancien. In: Briend J, Humbert J-B, editors. Tell Keisan (1971-1976) une cité phénicienne en Galilée. Fribourg/Göttingen: Vandenhoeck & Ruprecht; 1980. p. 361-78.

207. Liphschitz N. The archaeobotanical finds. In: Ben-Tor A, Zarzecki-Peleg A, Cohen-Anidjan S, editors. Yoqneʿam - 2: The Iron Age and the Persian period: final report of the archaeological excavations (1977 - 1988). Jerusalem: The Hebrew University of Jerusalem and the Israel Exploration Society; 2005. p. 395-9.

208. Gilliland DR. Paleoethnobotany and paleoenvironment. In: LaBianca S, Lacelle L, editors. Hesban 2 Environmental foundations. Berrien Springs: Andrews University Press; 1986. p. 123-42.

209. Farahani A, Porter BW, Huynh H, Routledge B. Crop storage and animal husbandry at Early Iron Age Khirbat al-Mudayna al-‘Aliya (Jordan): A Paleoethnobotanical Approach. In: McGeough KM, editor. The archaeology of agro-pastoralist economies in Jordan. The Annual of the American Schools of Oriental Research. 69. Boston: American Schools of Oriental Research; 2016.

210. Engel T. Charcoal remains from an Iron Age copper smelting heap at Feinan, Wadi Arabah (Jordan). Vegetation History and Archaeobotany 1993;2:205-11.

211. Kislev ME. Ancient infested wheat and horsebean from Horbat Rosh Zayit. In: Gal Z, Alexandre Y, editors. Horbat Rosh Zayit, an Iron Age storage fort and village. Jerusalem: Israel Antiquities Authority; 2000. p. 206-20.

212. Baruch U, Liphschitz N. Appendix 2: Charred wood remains. In: Gal Z, Alexandre Y, editors. Horbat Rosh Zayit: An Iron Age Storage Fort and Village, vol 8 Jerusalem: Israel Antiquities Authority; 2000. p. 203–5.

213. Crawford P. Botanical remains. In: Stone EC, Zimansky PE, editors. The Iron Age settlement at 'Ain Dara, Syria Survey and soundings. Oxford: British Archaeological Reports 1999. p. 113 - 21.

214. Orendi A, Smejda L, McKinny C, Cassuto D, Sharp C, Shai I. The agricultural landscape of Tel Burna: Ecology and economy of a Bronze Age/Iron Age settlement in the southern Levant. Journal of Landscape Ecology 2018;10(3):165-88.

215. Liphschitz N, Waisel Y. Analysis of the botanical material of the 1969-1970 seasons and the climatic history of the Beer-Sheba region. In: Aharoni Y, editor. Beer-Sheba I Excavations at Tel Beer-Sheba 1969-1971 seasons. Tel Aviv: Tel Aviv University; 1973. p. 97-105.

216. Badura M, Rzeźnicka E, Wicenciak U, Waliszewski T. Plant remains from Jiyeh/Porphyreon, Lebanon (seasons 2009–2014): preliminary results of archeobotanical analysis and implications for future research. Polish Archaeology in the Mediterranean. 2016;25:487-510.

217. Miller NF. Plant remains from Ville Royale II, Susa. Cahiers de la Délégation Archéologique Française en Iran 1981;12:137-42.

218. Simchoni O, Kislev ME. Charred by-products of olive-oil production in the Iron Age. In: Mazar A, editor. Excavations at Tel Beth-Shean Vol I. Jerusalem: The Hebrew University of Jerusalem 2006. p. 679-86.

219. Liphschitz N. Chapter 13. The botanical remains. In: Meshel Z, editor. Kuntillet 'Ajrud (Horvat Teman) An Iron Age II religious site on the Juda. Jerusalem: Israel Exploration Society; 2012. p. 342-50.

220. Rovira N. Les graines et les fruits. In: Aubet S, Eugenia M, Núñez FJ, Trellisó L, Abillamaa R, editors. The Phoenician cemetery of Tyre-Al Bass II Archaeological seasons 2002-2005. Bulletin d'archéologie et d'architecture libanaise. 9. Beyrouth: Direction Générale des Antiquités; 2014. p. 487–501.

221. Çizer Ö. Archaeobotanical macro remains from Late Bronze Age Kinet Höyük and Tell Atchana (Alalakh) in southern Turkey: Economical and environmental considerations. Master Thesis. Tübingen: University of Tübingen; 2006.

222. Riehl S, Deckers K. Vorbericht zu einigen eisenzeitlichen und mittelalterlichen Pflanzenreste vom Tell Halaf. In: Baghdo AH, Martin L, Novák M, Orthmann W, editors. Tell Halaf: Vorberichte über die erste und zweite syrisch-deutsche Grabungskampagne. Wiesbaden: Harrassowitz; 2009. p. 105-18.

223. Liphschitz N, Waisel Y. Analysis of the botanical material of the 1975-6 seasons of Tell Qiri. In: Ben Tor A, Portugali YA, editors. Tell Qiri, a village in the Jezreel Valley. Qedem. Monographs of the Institute of Archaeology 24. Jerusalem: The Hebrew University of Jerusalem; 1987. p. 252-6.

224. Greenfield T, Rosenzweig M. Assyrian provincial life: a comparison of botanical and faunal remains from Tušhan (Ziyaret Tepe), Southeastern Turkey. In: Stucky R, Kaelin O, Mathy H-P, editors. Proceedings of the 9th International Congress on the Archaeology of the Ancient Near East, Volume 2. Wiesbaden: Harrassowitz; 2016. p. 305-21.

225. Hopf M, Willerding U. Pflanzenreste. In: Kleis W, editor. Bastam II Ausgrabungen in den Urartäischen Anlagen 1977-1978. Berlin: Gebr. Mann; 1988. p. 263-317, plates 44-46.

226. Selmeier A. X. Holzfunde. In: Kleis W, editor. Bastam II Ausgrabungen in den Urartäischen Anlagen 1977-1978. Berlin: Gebr. Mann; 1988. p. 319-26, plates 47-48.

227. van der Steen EVD, Yassine K. Tell el-Mazar II: Excavations on the Mound 1977-1981. Field I. Oxford: British Archaeological Reports; 2012.

228. Oybak Dönmez E. Urartian crop plant remains from Patnos (Agri), eastern Turkey. Anatolian Studies 2018;53:89-95.

229. Liphschitz N. Botanical remains. In: Beit-Arieh I, editor. Tel ‘Ira – a Stronghold in the Biblical Negev. 15. Tel Aviv: Tel Aviv University; 1999. p. 476-9.

230. Orendi A, Deckers K. Agricultural resources on the coastal plain of Sidon during the Late Iron Age: archaeobotanical investigations at Phoenician Tell el-Burak, Lebanon Vegetation History and Archaeobotany 2018:717-36.

231. Weiss E, Kislev ME, Mahler-Slasky YP. Plant remains. In: Stager LE, Master DM, Schloen JD, editors. Ashkelon 3 The seventh century BC Winona Lake: Penn State University Press; 2011. p. 591-613.

232. Kyllo MA, Hubbard RNLB. Median and Parthian plant remains from Tepe Nush-i Jan. Iran. 1981;19:91-100.
